# Contributions of low- and high-level contextual mechanisms to human face perception

**DOI:** 10.1371/journal.pone.0285255

**Published:** 2023-05-02

**Authors:** Mehmet Umut Canoluk, Pieter Moors, Valerie Goffaux

**Affiliations:** 1 Research Institute for Psychological Science (IPSY), UCLouvain, Louvain-la-Neuve, Belgium; 2 Department of Brain and Cognition, Laboratory of Experimental Psychology, KU Leuven, Leuven, Belgium; 3 Department of Cognitive Neuroscience, Faculty of Psychology and Neuroscience, Maastricht University, Maastricht, The Netherlands; 4 Institute of Neuroscience (IoNS), UCLouvain, Louvain-la-Neuve, Belgium; Federal University of Paraiba, BRAZIL

## Abstract

Contextual modulations at primary stages of visual processing depend on the strength of local input. Contextual modulations at high-level stages of (face) processing show a similar dependence to local input strength. Namely, the discriminability of a facial feature determines the amount of influence of the face context on that feature. How high-level contextual modulations emerge from primary mechanisms is unclear due to the scarcity of empirical research systematically addressing the functional link between the two. We tested (62) young adults’ ability to process local input independent of the context using contrast detection and (upright and inverted) morphed facial feature matching tasks. We first investigated contextual modulation magnitudes across tasks to address their shared variance. A second analysis focused on the profile of performance across contextual conditions. In upright eye matching and contrast detection tasks, contextual modulations only correlated at the level of their profile (averaged Fisher-Z transformed r = 1.18, *BF*_*10*_ > 100), but not magnitude (*r* = .15, *BF*_*10*_ *=* .61), suggesting the functional independence but similar working principles of the mechanisms involved. Both the profile (averaged Fisher-Z transformed *r* = .32, *BF*_*10*_ *=* 9.7) and magnitude (*r =* .28, *BF*_*10*_ *=* 4.58) of the contextual modulations correlated between inverted eye matching and contrast detection tasks. Our results suggest that non-face-specialized high-level contextual mechanisms (inverted faces) work in connection to primary contextual mechanisms, but that the engagement of face-specialized mechanisms for upright faces obscures this connection. Such combined study of low- and high-level contextual modulations sheds new light on the functional relationship between different levels of the visual processing hierarchy, and thus on its functional organization.

## 1. Introduction

Evolution and lifespan experience equip biological systems with the sensory means to interact efficiently with their natural environment [1, 2]. One of the most crucial challenges for humans is to process the dense array of visual information transmitted by the faces of their conspecifics (e.g., familiarity, individual identity, emotional expression, speech movements, physical health [3, 4]). A hallmark of human face perception resides in the robust influence of context on the processing of the local facial features (e.g., eyes, nose, or mouth). For example, humans discriminate the local features more accurately when embedded in a face than presented in isolation or in an atypical (e.g., scrambled) face context [5–10]. In the composite face and congruency paradigms, the observer’s sensitivity (in terms of accuracy, d’, and/or reaction times) to a local feature decreases if it is artificially inserted into a face context of a different similarity status (in case the observer performs a discrimination task), or of a different identity (in the case of a recognition task) from the local target [6, 11–19].

Susceptibility to contextual modulation is a fundamental functional property of human face processing; it is thought to enable the fast and efficient processing of the whole stimulus for the accurate identification of faces despite their high visual similarity [20–24]. Surround influences on local feature processing are thought to be part of what makes face processing special, as they are systematically stronger for faces than for other visual categories [25, 26]. Consistent with this notion of specialization, the mechanisms underlying face contextual modulations significantly recede when the face stimulus is turned upside down. The selective vulnerability of face contextual modulations to a reversible image manipulation such as inversion [5, 6, 27–31] suggests a functional locus in the high-level visual cortex tuned to the natural statistics of the upright face (i.e. in the fusiform gyrus [16, 32–35]; see also [36–42] for the contribution of amygdala, parietal, and superior temporal cortex to face processing). As a result, research on contextual modulations for faces has developed as a relatively independent field with its own terminology (‘holistic’ or ‘configural’ processing) and paradigms (cf. [43]).

Yet, while faces trigger particularly strong contextual modulations, these are observed for most types of stimuli, across the sensory systems of many species, and have been documented using a wide variety of techniques (from cellular electrophysiological recordings [44–48] to psychophysics [49–52]). Contextual modulations are observed from the earliest levels of sensory processing, in retinal ganglion cells, the lateral geniculate nuclei [53–56], as well as in the primary visual cortex (V1; [45, 48, 57–59]), extrastriate cortex (V2 [60], V4 [61], MT [62]), and inferotemporal cortex [63, 64].

Contextual modulations are proposed to be critical for the representation of contours, corners, local curvature as well as for figure-ground segmentation [65–71]. Vinje & Gallant [2, 71] showed that contextual modulations increase the sparseness of neural responses (i.e., a sparse neural response is when a few narrowly-tuned neurons are active at any moment; see also [72–74]. By making the neural code sparser, contextual modulations are thought to improve metabolic efficiency, feature selectivity as well as information transmission in the visual system, for an optimal coding of sensory information [75].

Given the functional importance and pervasiveness of contextual modulations in vision, it seems unlikely that contextual modulations for upright faces are exclusively mediated by high-level category-specialized mechanisms. A first indication comes from the observation that contextual modulations are not mandatory for upright faces; indeed their engagement depends on so-called basic image properties such as spatial frequency, and orientation content ([13, 76–81]; but see [82]). The strength of contextual influence in low-level stimuli is similarly influenced by spatial frequency, orientation content and contrast [75]. Another indication that contextual mechanisms for basic and face stimuli may be tightly linked resides in their common dependence on the strength of the local input. At low-level stages of visual processing, contextual modulations are most extensive when local visual input is weak (due to e.g., low contrast or ambiguity; evidence in retinal ganglion cells, the Lateral Geniculate Nuclei: [55]; primary visual cortex: [45, 48, 83], area MT: [84, 85]. A similar phenomenon rules the contextual modulations for faces. Goffaux (2012) [14] demonstrated that, when human observers discriminate a local face feature, the strength of contextual modulations decreases as a function of local feature dissimilarity. In other words, the more dissimilar the feature characteristics, the less influence the context has on the processing of a given feature ([16, 86–88]; see also [12, 89–92] for indirect evidence).

These results suggest that contextual modulations at the general low-level and specialized high-level stages of processing are governed by the same principle: when the local sensory signals carried by a given stimulus are weak, the visual system integrates information across a larger portion of visual space. The evidence for this shared principle comes from studies that focus on a given processing level (e.g., only low, or high; [14, 44, 45, 48, 75]), and the functional link of contextual modulations at low- and high-levels of visual processing level has yet to be determined. We take this functional commonality as potentially reflecting the influence of general low-level mechanisms on holistic face processing, and address it systematically across levels using an interindividual differences approach. It is important to note that our aim here is not to question the specialization of contextual mechanisms for the processing of the upright face; we cast no doubt on the rich and consistent evidence that this category recruits special contextual mechanisms at high-level stages of processing. Rather, our aim is to examine the extent of this specialization, namely the inter-individual variability in face contextual modulations (i.e., holistic/interactive processing abilities; [93–95]) that is explained by general low-level mechanisms (see [96]). An alternative explanation to their common susceptibility to local input strength is that low- and high-level contextual modulations involve comparable but independent principles (see [62]).

The present study addresses these questions by testing the same group of participants with experimental paradigms tapping into the susceptibility of contextual modulations to local input strength in a basic and a complex face task. In the basic, contrast detection task, participants detected the presence of a grating; in the complex face task, they discriminated a local facial feature (i.e., the eye region) in pairs of faces. We parametrically manipulated the strength of the local input by modulating the target grating’s contrast in the contrast detection task and the amount of physical difference in the eye region using morphing in the eye matching task (as in [14]). The influence of context was investigated by presenting the local target (grating or eye region) in isolation, in same context (isotropic contextual grating or two identical face contexts) or in different context(s) (orthogonal contextual grating or two different face contexts). The contextual modulations in the eye matching task were investigated using upright and inverted faces. Inverted face stimuli are as complex as upright faces but recruit less specialized high-level mechanisms (e.g. [27, 30, 97]). Inversion was found to weaken both the magnitude of face contextual modulations (e.g., [27, 87, 98]) and its relationship to local input strength [14]. Therefore, face inversion allows studying the susceptibility of contextual modulations to local input strength at high-level but non-specialized processing stages.

Across tasks, we evaluated the influence of context on a similar metric, namely the target signal strength necessary to reach threshold performance and analyzed the relationship of contextual modulation magnitudes across tasks in order to quantify the functional link between low- and high-level contextual mechanisms. By analyzing the relative *profile* of input strength thresholds across context conditions in each task, we further investigated potential qualitative similarities in the way contextual modulations emerge at low- and high-level (non-)specialized stages of visual processing. The (absence of a) correlation in contextual modulation magnitudes would suggest functional (in)dependence of these mechanisms, whereas a(n absence of) correlation in profiles would indicate that low- and high-level contextual mechanisms are governed by similar (independent) principles. A significant correlation in both the magnitudes and profiles would indicate that low-level mechanisms contribute to the high-level contextual processing.

## 2. Methods

### 2.1. Participants

Sixty-two participants (psychology students) from UCLouvain, Belgium (50 females, mean age 21.8 ± 3.89 years, 6 left-handed) participated in the experiment in exchange for monetary reimbursement or class credits. Handedness was measured using Edinburgh Handedness Inventory [99]. Prior to the experiment, the Freiburg Visual Acuity Test (FrACT; [100]) was conducted for each participant to ensure their visual acuity was within the normal range. All participants had normal or corrected-to-normal vision (mean acuity of 1.6 ± .27 dec, mean logMAR = -.19 ±.09). Additionally, facial recognition abilities were tested using a computerized version of the Benton Facial Recognition Test (BFRT; [101, 102]). All participants performed above or at the borderline of a normal score of 39/54 [103] (mean score: 45/54 ± 3.44). The experimental protocol adhered to the Declaration of Helsinki and was approved by the ethical committee of the UCLouvain (approval number: 2016/13SEP/393). Prior to participating in the study, all participants provided informed consent. Three participants could not reach the required performance level in the eye matching task’s practice session (65% accuracy) and were removed from the experiment. Remaining 59 participants (47 females, mean age 21.83 ± 3.85 years, 6 left-handed) did both tasks.

### 2.2. Stimuli

Participants performed both tasks seated in a dark room (whose walls are painted black to ensure the lux/cd ^ m2 are lowest when the doors are closed) with their head position restrained by a chinrest, at a distance of 57 cm from the computer monitor. Prior to starting, all participants were dark adapted for at least 30 seconds. We used a 27” Dell LCD monitor with a spatial resolution of 1920 x 1080 pixels, a temporal resolution of 60 Hz, and a maximum luminance of 100 cd/m^2^. Monitor luminance levels were linearized using a ColorCAL MKII colorimeter **(**Cambridge Research Systems, Kent, UK). Luminance levels, gamma correction and the temporal resolution of the monitor were periodically controlled to ensure the same values were kept constant throughout the experiment. Both tasks were run on PsychoPy (2020.2.4post1; [104]) and Python 3.8.

In the eye matching task, we presented pairs of stimuli (either full face or isolated eye regions). Models on the photographs were young adult (psychology) students and alumni (aged 18–25 years) of the UCLouvain, who gave written consent for the use of their image [105]. In a given pair, the dissimilarity of the local face region relevant to the task (i.e., eyes and brows) was manipulated parametrically using morphing (similar to [14], see below for a detailed description).

In order to generate the different levels of feature dissimilarity, morphs were created using 32 full-frontal grayscale pictures (on a scale from 0 to 1) of unfamiliar white neutral faces from each gender without glasses, facial hair, or makeup. We morphed 16 pairs of same-gender faces (eight pairs for each gender). Prior to morphing, the luminance and root-mean-square (RMS) contrast of all face images were set to a mean of .52 and .12, respectively. Next, we ensured that the eye regions of morphed faces were comparable in terms of inter-pupillary distance and physical similarity and warranted a smooth morphing within pairs by measuring inter-pupillary distance in each face image, and pairing the faces with comparable values (average pixel difference = 1.32 ± 1.31 pixels). After this initial pairing, pixelwise similarity within the eye region was calculated within each pair using *corr2* function. The pixelwise similarity of the target eye region was .8 on average (SD = .014). For each gender, the eight pairs lying within two standard deviations [106] around the mean pixelwise similarity distribution were used for morphing; the remaining eight pairs were not morphed and used as “face contexts”. An additional set of 12 female faces (six pairs of female faces) which had undergone the same procedure were used for practice trials. These image manipulations and calculations to select optimal pairings were performed using MATLAB (Natick, MA).

**Fig 1:**
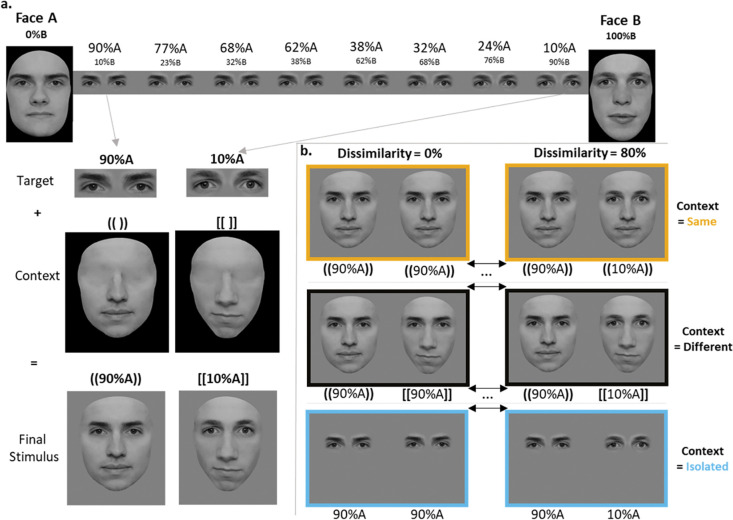
Eye matching task stimuli and conditions. **a.** Morphing procedure and stimulus creation. Two morphed eye regions are selected from the morphing continuum and pasted on two distinct face contexts. **b.** Same, Different and Isolated context conditions. Bracket shape (round, square) defines distinct face contexts, percentage values define morphing percentage in the eye region. Target regions within a pair had 0%, 24%, 36%, 53% or 80% dissimilarity. It can be seen here that when the target regions are the same (0% dissimilarity) and context differs, the contextual modulations are stronger compared to when target regions differ largely (80% dissimilarity).

We created morphing continua between the two faces of all pre-selected pairs using Morpheus Photo Morpher 3.17 (Morpheus Development, LLC, 2020). For each face, ~110 control points were used for morphing (contour: ~30 points, mouth: ~15 points, eyes and eyebrows: ~25 points each, and nose: ~15 points; [14, 107]). In each continuum, we selected faces at eight morphing levels: 90% of Face A (10% of Face B), 77%A (23%B), 68%A (32%B), 62%A (38%B), 38%A (62%B), 32%A (68%B), 24%A (76%B), 10%A (90%B). These levels were selected specifically so that for each non-zero dissimilarity level in the task (24%, 36%, 53% and 80%), all pairs crossed the midpoint (50%) of the morphing continuum. We extracted the target region (eyes and brows) of each selected morphed pair, and blended it onto two unmorphed “face contexts” whose eye areas were *a priori* replaced with a smooth skin texture (using Adobe Photoshop CS., Berkeley, CA; see **Fig 1A**). Since all stimuli were artificially generated, all were subject to same potential amount of artifacts across conditions.

Each face subtended a visual angle of ~7° in height, and ~5.2° in width. This size corresponds to the size of a human face seen at an average social distance (a human face is approximately of 18 cm in height and social distance is of 1.5 m approximately, e.g. [108]). The target eye region subtended a visual angle of ~1.4° in height, and ~4.1° in width. Center of the face image was 1.18° below the screen center in Upright trials, and 1.18° above in Inverted trials to ensure that the target eye region aligned with fixation. Stimuli were shown in their canonical Upright (0° rotation) or Inverted (180° rotation) position.

We additionally created noise masks by iteratively phase-scrambling each face image in Fourier space (in 500 iterations; see [109–111]. Iterative scrambling is used to prevent noise masks from being contaminated by uniform background pixels, and thus ensures that the spectral properties of the stimulus and mask are similar.

**Fig 2:**
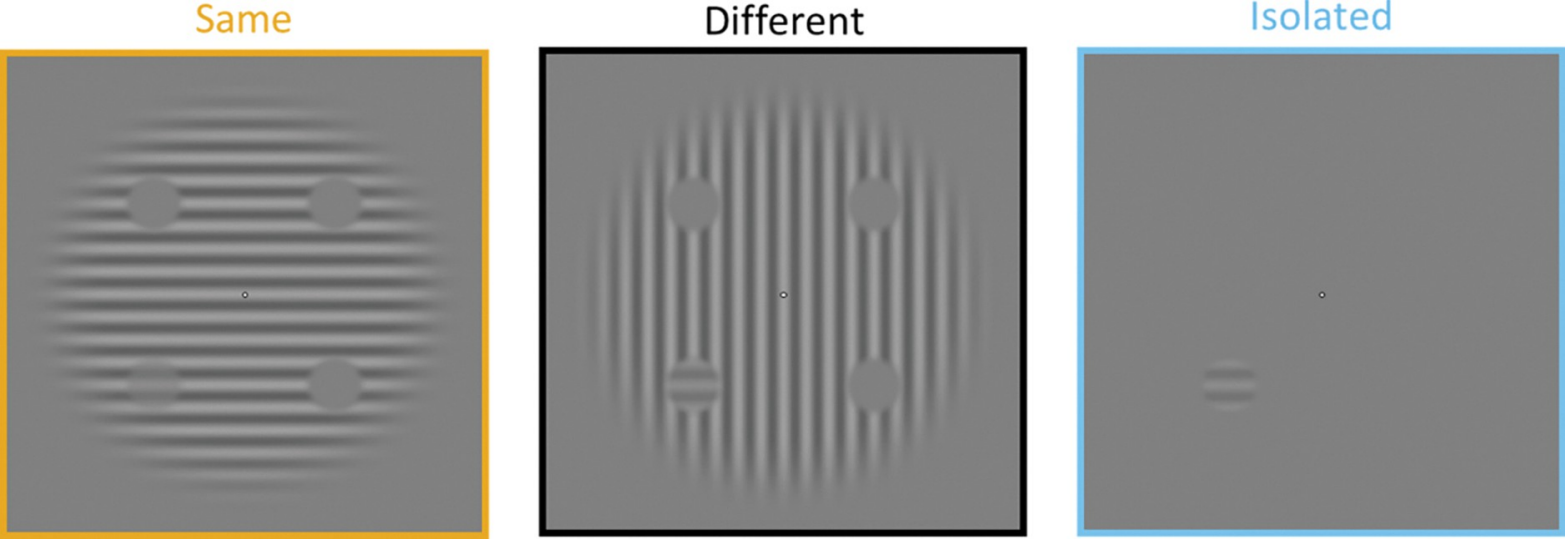
Same, different, and isolated context conditions of the contrast detection task. Participants were asked to locate the horizontal grating appearing in one of the four target regions. It can be seen here that although target contrast is identical in all three examples, target appears to be less visible in Same condition compared to other two conditions.

The stimuli and procedure of the contrast detection task were adapted from [112]. Stimuli consisted of four circular target regions (3° of visual angle each) in a circular context (diameter of 20°) (see **Fig 2**). Both the context and the target had a spatial frequency of 1 cycle per degree. The context was made up of a horizontally or vertically oriented grating with a contrast of 25%. Target regions were positioned at 5° in eccentricity, one in each quadrant of the visual field (at 45°, 135°, 225°, 315° from the horizontal axis).

On each trial, a horizontal grating with the same spatial frequency and phase as the context and with a variable, non-zero contrast was presented in one of the four target regions. The contrast of both the context and target regions ramped smoothly to zero at the edges with the use of a raised cosine filter. Additionally, a small fixation dot (diameter of 0.25°) was always present at the center of the screen during the task. The contrast of the target grating was determined using a Psi adaptive staircase procedure ([113]; for details, see [112]), which used a pool of 350 logarithmically spaced contrast values ranging from 0.001 to 1. There were two separate, interleaved staircases for each context condition. This interleaving procedure ensured that the algorithm did not settle into a narrow range of contrast levels within a condition.

### 2.3. Procedure

The experiment consisted of three different sessions of ~45 minutes each. The eye matching task was relatively long, and was divided into two equal sessions. The participants performed the eye matching task on the first and third sessions, and the contrast detection task on the second. This task order aimed at keeping participants motivated by alternating tasks between sessions. Participants took a break of at least 10 minutes between sessions.

**Fig 3:**
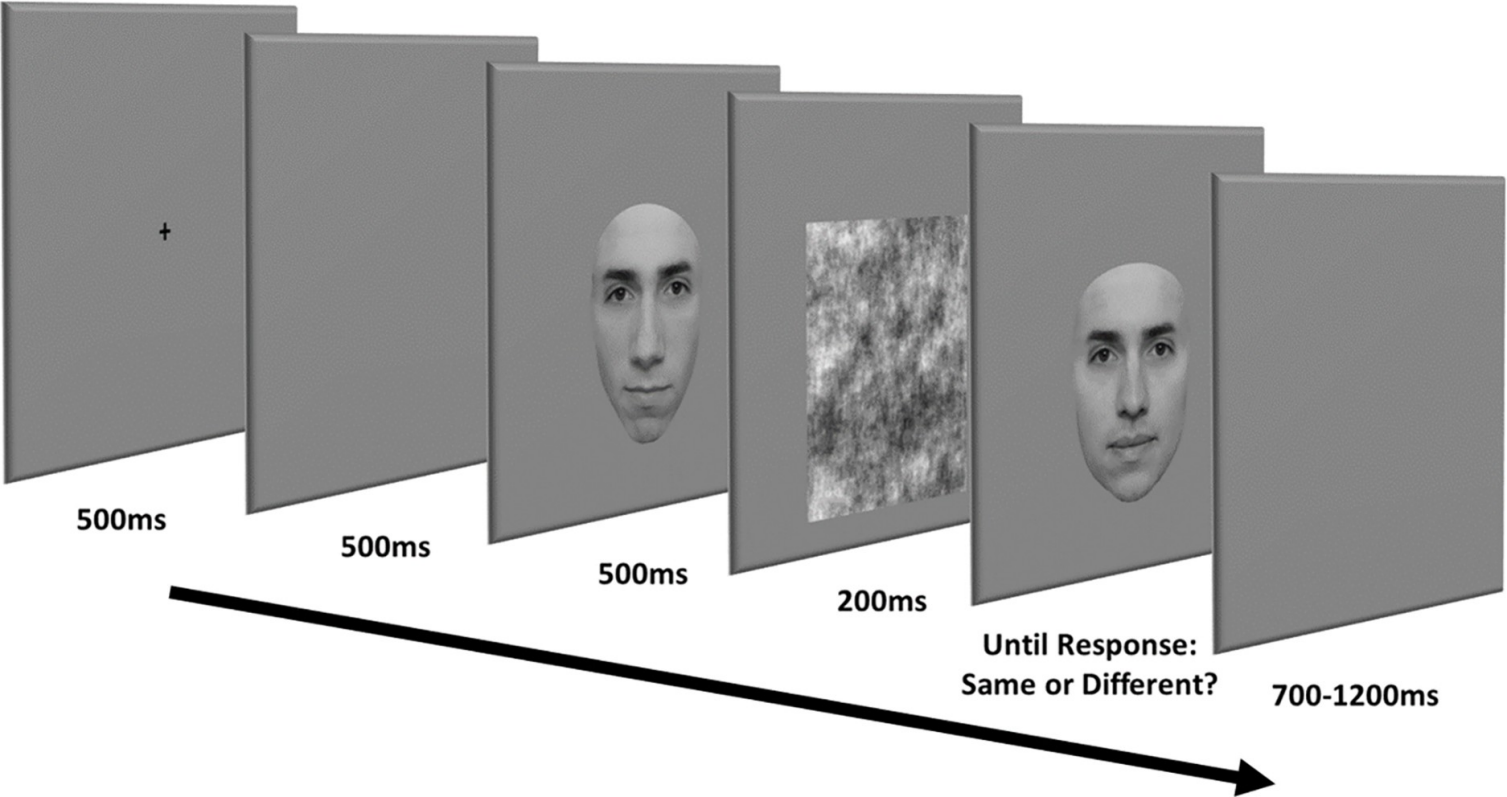
Eye matching task trial sequence. An example of 0% eye region dissimilarity and Different context condition trial. The participant sees a fixation cross for 500 ms at the beginning of each trial, followed by a blank screen. Afterwards, participant sees the first stimulus for 500 ms, then the noise mask for 200 ms, and finally, the second stimulus. The second stimulus stays on the screen until the participant indicates whether the eye regions were “Same” or “Different” between the two stimuli by button press, followed by a blank screen before the next trial starts.

In the eye matching task, a pair of faces (or eye regions) was presented successively in the center of the screen. Participants had to focus only on the eye region, ignore the rest of the face (when there was one), and determine whether the two eye regions shown in succession were strictly identical or differed in some way (i.e., two alternative forced choice). On each trial, participants first saw a fixation cross on a gray background for 500 ms, followed by a 500 ms blank screen and the first face for 500ms. The phase-scrambled version of the first face was then presented for 200 ms as a mask, directly followed by the second face which stayed on the screen until the participant gave a “Same” or “Different” response by button press (see **Fig 3**). After response, a blank screen was shown for an interval of random duration (ranging from 700 to 1200 ms) before the next trial started.

The eye matching task employed the method of constant stimuli with constant morphing dissimilarity values and was designed to obtain thresholds expressed in morphing units. This task consisted of 30 conditions: two orientations (Upright, Inverted) by three context conditions (Same, Different, Isolated) by five dissimilarity levels (0%, 24%, 36%, 53%, 80%). The 24%, 36%, 53% or 80% dissimilarity trials called for”Different” response; they were presented 16 times in each orientation and context condition (384 trials in total). In order to balance the responses, we prepared 384 “Same” response trials made out of 64 0% dissimilarity trials in each orientation and context condition. In “Same” response trials, 10%, 32%, 62% or 77% target morphs were repeated twice. The 24% dissimilarity trials comprised 38% and 62% morphs, the 36% dissimilarity trials comprised 32% and 68% morphs, the 53% dissimilarity trials comprised 24% and 77% morphs, and the 80% dissimilarity trials comprised 10% and 90% morphs. Doing so, we ascertained that half of the morphs used in the non-zero dissimilarity conditions appeared in the 0% dissimilarity condition. In total, there were 768 trials in the eye matching task.

Independent variables in this task were five dissimilarity levels (0%, 24%, 36%, 53%, 80%), three context conditions (same, different, isolated) and two orientations (upright, inverted). A trial was made up of a combination of a level of each of these independent variables. Our dependent variable was the (proportion of) “different” responses given by a participant. In 0% dissimilarity trials, each unique target (i.e., a specific eye region at a specific morph level) appeared six times. In other dissimilarity levels, each unique target appeared three times. We replicated trials across Upright and Inverted conditions.

Each experimental session of the eye matching task was divided into eight blocks of 48 trials. Within each block, there were equal number of “Same” (0% dissimilarity level, 24 trials) and “Different” (24%, 36%, 53% or 80% dissimilarity levels, six trials each) response trials equally distributed across contexts and orientation conditions, in randomized order. If a participant performed below 65% on a given block, a text appeared on a red background, suggesting them to take a longer rest, and stay attentive. This was merely a notification and did not impact the experimental design in any way. It was used to inform the participant about their performance, and to keep them attentive and active during the experiment. Instructions emphasized accuracy over speed. Participants were required to do a short practice round (four short blocks of 24 trials) before each session to familiarize with the task. If necessary, participants re-took the practice round, until they reached a minimum of 65% correct response accuracy. The practice data was not included in any further analyses, and this 65% correct response criterion was only used as a prerequisite to start the experiment.

In the contrast detection task, the target horizontal grating appeared in three distinct context conditions: in Isolation, in an iso-oriented (“Same”) context, and in an orthogonal (“Different”) context. As in Mannion et al., [112] each context condition was presented in separate blocks. Each block consisted of 80 trials, made up of two staircases of 40 trials. There were three block repetitions per condition, i.e. a total of 9 blocks (720 trials in total). Block order was randomized across participants, with successive blocks belonging to distinct conditions. Each trial started with a preparatory, black fixation dot of 500ms. The fixation dot then turned to white for another 50ms. Next, the white fixation dot and the target stimulus (embedded in a context or presented in isolation, depending on the condition) was presented in one of the four possible target regions for 100ms. After the stimulus presentation, the fixation dot turned to grey to signal the participant that they could give their response. The target could potentially appear in any of the four target regions as in [112], but we asked the participant to report only the side on which it appeared (left or right) so that both tasks involved a 2-AFC. As in the eye matching task, accuracy was emphasized over speed. The contrast detection task was relatively simpler, so the participants were asked only to perform a brief practice round of 10 trials before the experiment to ensure they understood the task, and the data from the practice were not included in any of the analyses.

### 2.4. Analyses

The purpose of the study was to investigate the functional dependence and similarity in working principles between low- and high-level contextual modulations. We first quantified the shared inter-individual variance in contextual modulation magnitude across low- and high-level mechanisms. A second analysis focused on profiles, examining whether these two mechanisms showed similar characteristics. All data were visualized using the *ggplot2* package in R [114].

Across tasks, we first investigated the function relating performance to local input strength (eye region dissimilarity or grating contrast). Since the definition of local input strength varied between tasks (i.e., 5 levels of target dissimilarity (0%, 24%, 36%, 53%, 80%) in the eye matching task; 350 levels of contrast (between 0.001 and 1) in the contrast detection task), we z-transformed the local input strength values for the eye contrast task, and log10 and z-transformed them for the contrast detection task to bring the two into a comparable scale. After these transformations, local visual input strength was within the range of -3 to 3 for both tasks.

The z-scaled data from both tasks were submitted to a Bayesian generalized linear mixed model [115, 116] by means of *brms* package available in R [117, 118]. We chose a Bayesian approach to determine not only the existence of a correlation and the evidence for it, but also the strength of the evidence for a null correlation when/if it occurs. Using a mixed model makes it possible to include inter-individual variability in the statistical model where the estimates for the individuals are influenced by other individuals (due to the hierarchical nature of the model), yielding better estimates and conferring more statistical power to the analysis compared to other approaches (see [119] for more details).

**Fig 4:**
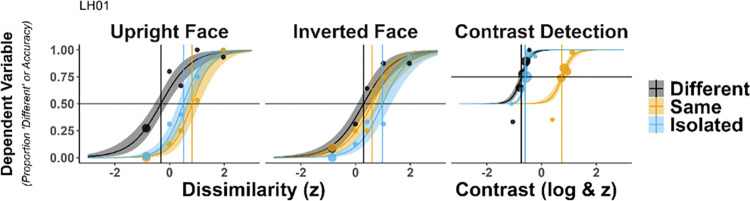
Data and psychometric functions of a representative participant. The dependent variable in the eye matching task is the proportion of ‘Different’ responses, and accuracy in the contrast detection task. Dots represent actual data (varying in size depending on frequency of presentation) and curves represent the model fits, with 95% HDI shaded ribbons. Vertical lines mark the “***threshold****”* for a given condition (at .5 in the eye matching task, at .75 for the contrast detection task).

The dependent variable in the contrast detection task was accuracy. Participants could give two responses: left or right. In such a 2-AFC detection task, the guess rate (i.e., lower asymptote of the psychometric function) is set at 0.5. The contrast value at .75 was taken as contrast detection “threshold”. In the 2-AFC eye matching task, participants could give a “Same” response or “Different” response. Here, the dependent variable was the proportion of “Different” responses instead of accuracy. The reason for that was that in this task, the outcome of interest was not how accurately a participant was telling whether the eyes were “Same” or”Different”, but how the point of subjective equality (PSE) was affected in response to varying contexts. The PSE in this task would indicate the amount of target feature dissimilarity (i.e., morphing difference) necessary to reach a rate of 50%”Different” responses. In this case, the guess rate was set at 0 and the PSE, or “threshold” was measured at .5 (see **Fig 4**).

The eye matching task consisted of two different orientation conditions: Upright and Inverted. We treated these two orientation conditions separately. With this adjustment, the factor, “task” has three different levels: “Upright Face”, “Inverted Face”, “Grating”.

We fitted a logit link function to model the sigmoidal pattern of the psychometric functions in each task (**Eq 1**). We put a normal distribution prior on all regression weights, and a lognormal distribution prior on the parameters capturing the random effects. All random effects were included, yet are assumed to be uncorrelated to each other (by means of || term in the formula) to increase sampling efficiency and ensure model convergence [118]. Doing so, we allow a participant to deviate from the average, but we don’t assume the size of these deviations to correlate across conditions [120]. Among several models tested, the model formula with the best fit was the one with complete interaction between all variables, and with random effect structure integrated (see S5 File for the details of model selection procedure):

$$\begin{array}{c} dependent\;variable∼.5*guess+(1-.5*guess)*inv\_logit(eta)\\ eta∼local\;input\;strength*task*condition+(local\;input\;strength*task*condition||participant) \end{array}$$ Eq 1

***brms* formula used to fit the sigmoidal functions.** The variable “guess” was set as “1” for the contrast detection task (0.5 guess rate) and “0” for eye matching task (0 guess rate).

All predictor variables were dummy-coded (as in [119]). Using these parameters and the formula, we ran four Markov chain Monte Carlo (MCMC) chains with 6,000 iterations each (3,000 of each were considered warm-up), resulting in 12,000 iterations after warmup. Default values were used for the *adapt_delta* and *max_treedepth* parameters. Running this model yielded MCMC chains that were converging according to Gelman-Rubin diagnostics such as effective sample size, the R-hat statistic, and the absence of divergent transitions.

After fitting the data from all tasks, we obtained the threshold value of the logit link function for each task and context condition, and for each participant using the formula, *threshold = − (intercept / slope)* as outlined in [121]. The local input strength threshold values were in terms of morphing units for the eye matching tasks, and in terms of contrast values in the contrast detection task. We compared these threshold values across tasks and contexts using the model’s estimates, and each threshold’s respective 95% Highest Density Interval (HDI; [122, 123]). We interpreted the differences across conditions by testing whether the HDI of the differences between conditions had an overlap with zero. A HDI that excludes zero indicates a “significant”, or a non-negligible difference across conditions.

In order to obtain a measure of the contextual modulation magnitude in each participant, we submitted the individual threshold values in the various context conditions of each task to a two-step regression of regressions analysis [24]. Each task comprised a control “Isolated” condition in which the target region (eyes and brows, or grating) was presented on its own. In a first regression, we regressed participants’ thresholds in the “Isolated” context condition from “Same” context and “Different” context thresholds, therefore removing all variance related to the control condition from the conditions where a face context was present. In the second regression, we regressed the residuals of the “Different” context condition from the residuals of the “Same” context condition. We chose to use this approach because the residuals of this two-step regression delivers an estimate of each individual’s contextual modulation magnitude that was not contaminated by the variance of the compared conditions, as opposed to simple subtraction method where the final outcome contains variance from both of the compared conditions [24].

We addressed whether contextual modulations for eye matching and grating contrast detection are functionally related by submitting the final product from the two-step regression to a Bayesian correlation analysis. We chose a Bayesian approach because of its ability to determine the strength of evidence presented by the data in supporting a particular hypothesis (H0 or H1), hence, its potential to interpret a finding, even when it is null. We computed Bayes Factors (BFs) of the correlation using BayesFactor package [124] in R. A BF_10_ value indicates the support for H1 (e.g., correlation) over H0 (e.g., no correlation) given the data (conversely, BF_01_ would indicate support for H0 over H1, and can be calculated with 1/BF_10_). We followed Jeffreys’ guidelines [125, 126] to interpret the BF values: a BF_10_ value of 1 would mean equal amount of evidence for both H0 and H1, and therefore inconclusive. A BF_10_ value between 1 and 3 is considered anecdotal evidence in favor of H1 over H0, whereas a BF_10_ between 3 and 10 indicates substantial evidence for H1. BF_10_ > 10, BF_10_ > 30 and BF_10_ > 100 are regarded as strong, very strong and decisive evidence for H1, respectively.

In an additional analysis, instead of regressing out threshold values stemming from the distinct context conditions to obtain a quantitative measure of contextual susceptibility, we investigated whether the modulation of thresholds across the three context conditions was qualitatively similar across tasks on an individual level. To this extent, 3-point vectors of thresholds from each individual (Isolated, Different, Same) were submitted to a Pearson correlation analysis across tasks. This two-dimensional (2D) correlation approach results in a single correlation value per comparison (Upright/Inverted, Upright/Contrast Detection, Inverted/ Contrast Detection) in each individual. These correlation values are then Fisher-Z transformed. Because transforming 1 (and -1) correlation coefficients yields extreme values, we set any correlations with a value of 1 (or -1) to .99 (or -.99) before the transformation. We obtained empirical chance levels by permuting 1000 times [127] the 3-point vectors in each task (so that the new ranks differed from initial order) and averaging the correlations of each iteration. Finally, we quantified the profile similarities by testing whether the sample of (Fisher-Z transformed) correlation coefficients differs from empirical chance level by using Bayesian one-sample t-tests (see [109] for a similar analysis). We report the HDI (to interpret whether they overlap with the empirical chance or not) and Bayes Factors of these one-sample t-tests.

## 3. Results

**Fig 5:**
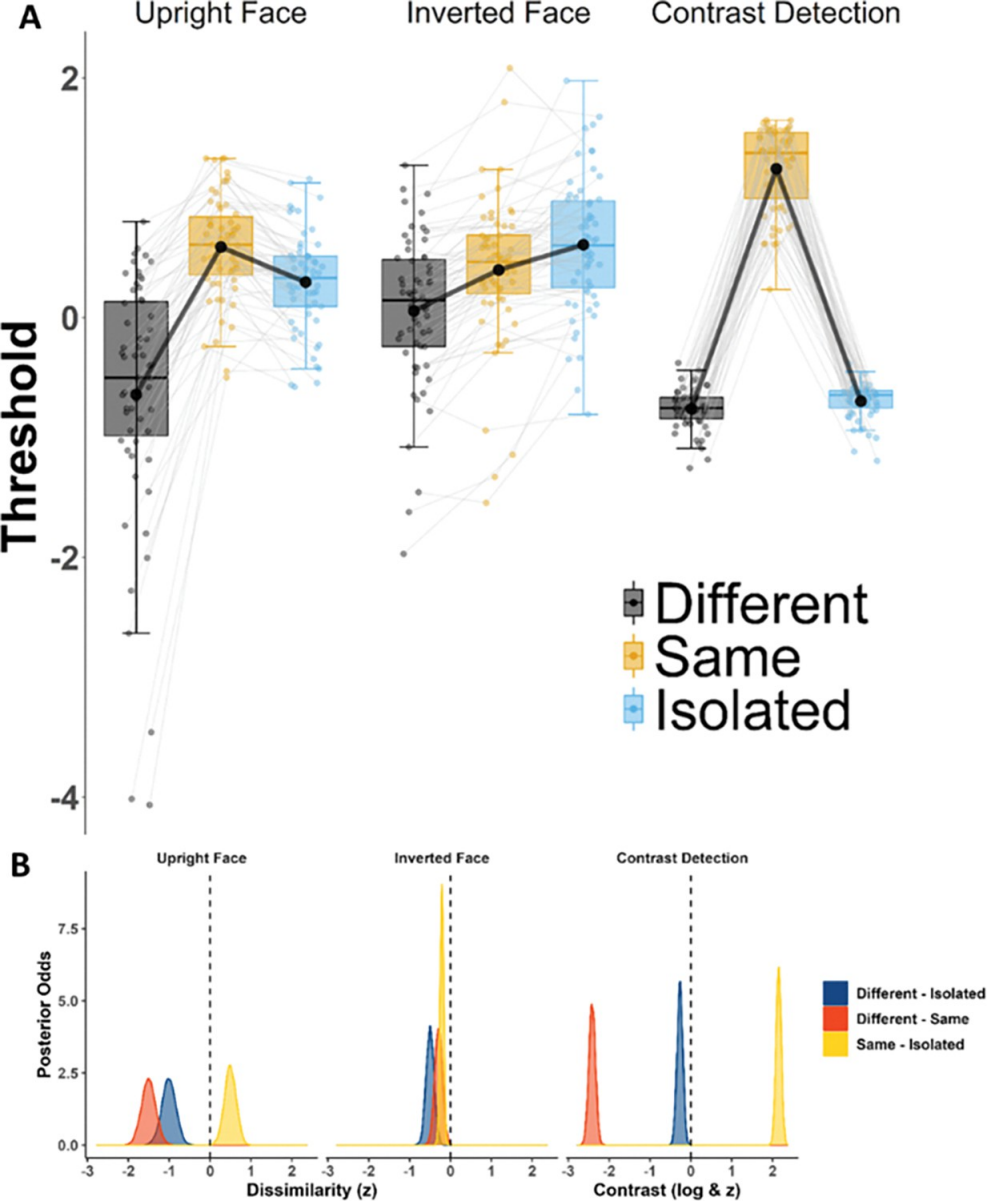
**A. Z-scaled threshold estimates in eye matching (left) and contrast detection (right) tasks**. Each dot represents the z-scaled threshold estimate for a given task and condition for a participant. The top, middle and bottom lines of boxes correspond to the first, second (median) and third quartiles (25^th^, 50^th^ and 75^th^ percentiles) respectively. The upper and lower whiskers extend until 1.5* Inter-Quartile Range. Faded grey lines connect the thresholds in respective conditions for a given participant. Bold black dots and lines display group means. **B. Posterior odds of differences across the three tasks**. The density plots display the posterior odds of the threshold differences. 95% interval of the distributions did not overlap with zero, therefore all differences were non-negligible.

In both tasks, contextual influences depended on local signal intensity, and were strongest when the local signal strength was low suggesting that similar functional principles may be at stake for both faces and gratings (see S1 File.; [14, 44, 45, 48]). Fig 5A illustrates the z-scaled threshold values for eye matching (Upright Face, Inverted Face) and contrast detection tasks. As expected, contrast detection thresholds increased for isotropic (same) oriented grating context, compared to an orthogonal (different) oriented grating context [83, 112, 128], and a similar mechanism was at play in the eye matching task: eye matching thresholds were higher when presented within a stable (same) face context, compared to different face contexts. As for the Isolated condition, its rank with respect to the Same and Different context conditions was similar in the contrast detection and Upright eye matching tasks, but differed in Inverted eye matching task. The threshold difference between Same and Different contexts was larger in the contrast detection task than in eye matching tasks (HDI [2.26, 2.57]). In the eye matching task, the threshold difference between Same and Different context was more pronounced for Upright Faces (HDI [1.18, 1.84]) compared to Inverted Faces (HDI [.10, .48]), corroborating past evidence that inversion attenuates contextual influences on the encoding of features.

While Same and Different context conditions rank similarly across tasks, the rank of the Isolated varied much more. In the contrast detection task, Isolated and Different thresholds were similarly low with only a mild advantage for Different context condition (HDI [-.13, -.40]), suggesting that the detection of local grating contrast works almost as effectively when the grating is inserted in an orthogonal context as when it is viewed in isolation. In the upright eye matching task, however, thresholds in the Isolated condition were closer to the Same than the Different context condition (HDI [.21, .77] and [-.67, -1.34], respectively). In the Inverted eye matching, the thresholds varied much less across context conditions than at upright, and culminated in the Isolated condition (HDI [-.12, -.30], [-.31, -.70] for Same-Isolated and Different-Isolated comparisons, respectively). This agrees with the proposal that inverted faces are processed more independently from context than upright faces. These similarities in the ranking of various context thresholds are systematically investigated later with the profile correlations below.

**Fig 6:**
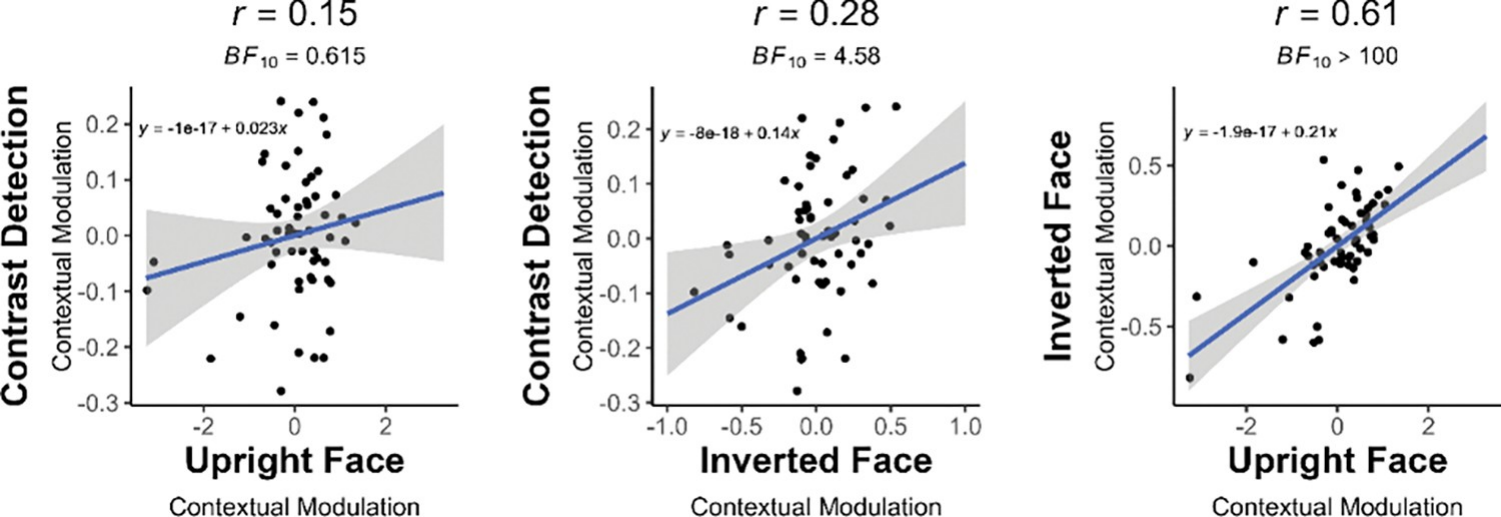
Correlation of contextual modulation magnitudes across tasks. Contextual modulation magnitude values are the product of *regression of regressions* method for each task. Blue line indicates the regression line and shaded area covers the standard error bounds.

In order to examine a possible functional link across low- and high-level (non)specialized contextual mechanisms, we first estimated the magnitude of contextual modulation by submitting the z-scaled individual thresholds to a regression of regression analysis. The residuals resulting from this analysis were then correlated across tasks (see Methods). This magnitude correlation served as a measurement of the quantitative relationship across tasks (see **Fig 6**).

We found anecdotal evidence for the absence of a correlation in contextual modulation magnitude between contrast detection and Upright eye matching (*r* = .15, *BF*_*10*_ *=* .61) suggesting the independence of the contextual mechanisms between low-level and high-level face-specialized processing. However, there was substantial evidence in favor of a weak correlation in contextual modulation magnitude between contrast detection and Inverted eye matching tasks (*r =* .28, *BF*_*10*_ *=* 4.58). This suggests that the contextual mechanisms involved in low-level stages can, to some extent, be tracked at high-level stages. Lastly, contextual modulation magnitude correlated strongly between Upright and Inverted eye matching, with decisive evidence in favor of a correlation (*r* = .61, *BF*_*10*_ *> 100*).

So far, we have focused on an aggregate metric of contextual modulation magnitude based on a regression of regression of the distinct context conditions. Considering the pervasiveness of contextual modulations in the visual system hierarchy, and their functional similarity across different processing levels, we now examine the profile of threshold variations across context conditions and investigate their qualitative similarity across tasks. As noted above, across tasks, thresholds increased when the target was presented within the Same context in comparison to Different context condition.

**Fig 7:**
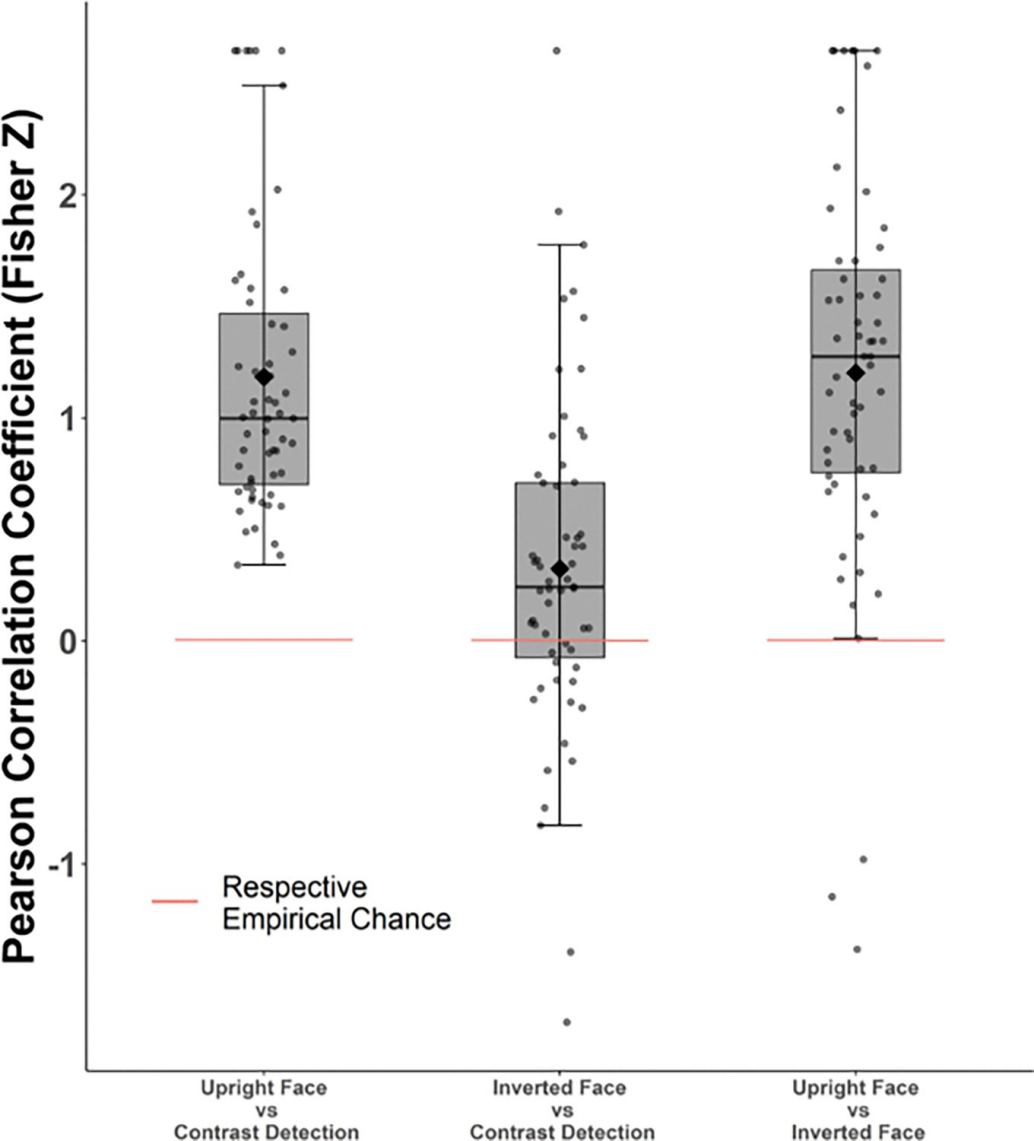
Correlation of the individual threshold profiles across tasks. Each dot represents a Fisher-Z transformed Pearson Correlation Coefficient value of an individual’s threshold values from the corresponding comparison. The top, middle and bottom lines of boxes correspond to the first, second (median) and third quartiles (25^th^, 50^th^ and 75^th^ percentiles) respectively. The upper and lower whiskers extend until 1.5* Inter-Quartile Range. Black diamonds display means.

The profile of threshold values indicates a similarity between contrast detection and Upright face tasks, while Inverted face profile seems to be standing out. In order to investigate the profile similarity more systematically, we analyzed the 2D correlation of threshold profiles across tasks at the individual level. We compared the sample of Fisher-Z transformed correlation values obtained from each task comparison against empirical chance level (i.e., correlation coefficient obtained by shuffled permutations) using Bayesian one-sample t-tests (see Analyses section for details). These analyses revealed conclusive evidence that the threshold profile in the Upright eye matching task correlates with the threshold profiles in the contrast detection task (averaged Fisher-Z transformed r = 1.18, HDI [1.02, 1.33], *BF*_*10*_ > 100 tested against empirical chance r = .005). Given the lack of a magnitude correlation between the two tasks, these findings suggest that the functional similarity in contextual processing between upright face eye matching and contrast detection is more qualitative than quantitative. We also observed conclusive evidence that the threshold profiles in Upright eye matching task correlates with Inverted eye matching task (averaged Fisher-Z transformed *r* = 1.20, HDI [.92, 1.40], *BF*_*10*_ > 100 tested against empirical chance r = .003; see **Fig 7**).

Inverted eye matching and contrast detection threshold profiles showed a relatively weaker profile similarity, but still with substantial evidence in favor of a correlation (averaged Fisher-Z transformed *r* = .32, HDI [.12, .48], *BF*_*10*_ = 9.7 tested against empirical chance r = .002). We would like to point out the correlations between Inverted Face and Contrast Detection tasks which are relatively smaller, both in terms of averaged Fisher-Z transformed r values and Bayes’ Factors, which suggests that this analysis (despite being only on the three points) is not blindly giving extremely large Bayes’ factors.

## 4. Discussion

Contextual modulations are an integral part of visual information processing [75]. They are ubiquitous in the visual processing hierarchy, and manifest a similar phenomenon at various levels: their impact on perception depends on the local input strength [14, 45, 48]. Despite extensive research endeavor to understand contextual modulations at distinct processing levels, little effort has been made to study potential relationships across levels (see [129] for a rare example). In this study, we investigated a potential link between low- and high-level contextual modulations through the lens of their dependence to local input strength. We reasoned that relying on this shared phenomenon across processing levels provides a rich metric that reflects a similar functional principle across tasks, and therefore increases the chance of observing a correlation, should there be one.

By testing the same group of participants in a low-level, contrast detection task and a high-level, eye matching task, we investigated contextual modulations separately for gratings, upright faces and inverted faces in the same individual observers. In the contrast detection task, as expected, we observed orientation-dependent contextual modulations as seen in [112]. Presenting a parallel-oriented context along with the target led to an increase in detection thresholds compared to presenting an orthogonal context or no context. We made our two tasks more similar by halving the number of response choices used in Mannion et al. [112] while keeping the four possible target locations, so the chance level differed across our and their studies (50% versus 25%). Despite this difference, we were able to replicate the results of Mannion et al. ([112]; see S1 File) and more particularly the strength and profile of contextual modulations were similar. In line with past evidence, inversion decreased the magnitude of face contextual modulations (e.g. [27, 130]). Additionally, our findings reaffirmed that inversion weakens the dependence of contextual modulations on the discriminability of local targets (see S1 File.; [14]). Given that the inverted face is as complex as the upright face and activates large portions of high-level visual cortices [30, 131–135], this stimulus condition adds an in-between layer that gives access to high-level but non-specialized processing.

We used a single Bayesian Hierarchical Model to obtain local input strength thresholds for each task. Utilizing a hierarchical model provided a stronger statistical power than running individual psychometric functions, mainly owing to its ability to employ variability of all subjects when making estimations about a parameter (in this case, the thresholds; [136]). Applying the *regression of regressions* method [24] to model the estimated thresholds across the three contexts provided us with a final metric of contextual modulation magnitude, which we compared across tasks. These quantitative analyses showed no relationship between low- and high-level face-specialized contextual modulation magnitude. Evidence for this absence of correlation was however anecdotal, suggesting that it may be due to the strong involvement of face-specialized mechanisms overriding low-level contextual mechanisms, and rendering them inconsequential, or unobservable in our behavioral measures. There was, however, substantial evidence for a weak relationship in the magnitude of low- and high-level non-face-specialized contextual modulations (i.e., between the contrast detection and inverted eye matching tasks). The fact that low-level contextual mechanisms explain a limited portion of the high-level non-specialized contextual modulations supports evidence for a functional link between simple and complex image contextual modulations. To our knowledge, our paper is the first one to demonstrate such dependence across the two. When the face-specialized mechanisms are engaged, this seems to cause a detachment in this dependence, leading to the absence of correlation between low-level and face-specialized contextual modulations.

Despite the disparity in their relationship to low-level mechanisms, we found that 37% of the variability in upright face contextual modulation is explained by contextual effects in inverted faces. Contextual modulations are expected to correlate more strongly within than across visual processing levels. Indeed, inverted faces still weakly activate face-specialized regions such as the FFA [16, 137, 138]. Yet, the large variability in upright face contextual modulation that remains to be accounted for after taking into account the variability in inverted face contextual modulations supports the functional specificity of the contextual mechanisms elicited by upright faces. This finding lends support to the existence of high-level mechanisms specialized for the natural statistics of the upright face (e.g. [18, 26, 139, 140]).

We found no relationship between upright face eye matching and contrast detection tasks in our quantitative analyses, however, their similar susceptibility to local input strength indicated that they may still share common functional principles. In an additional analysis, we examined the correlation of the profile of thresholds in Same, Different and Isolated conditions (i.e., qualitative relationship) across tasks. Whereas the magnitude analysis examined the functional dependence of contextual mechanisms across tasks, this profile analysis examines their similarity qualitatively, in terms of working principles. The profile of contextual modulation correlated positively across all three tasks suggesting they share some rules. As discussed in [75], and seen in various studies [14, 112, 128, 141, 142], contextual modulations are strongest when stimulus and context share similar properties. With dissimilar stimulus and context, contextual modulations are either weak, or facilitatory. This profile was replicated in our study, in all tasks. Despite the functional independence of upright face and contrast detection contextual mechanisms, this finding supports our hypothesis that comparable but independent principles govern low-level and face-specialized high-level contextual modulations. Inverted faces showed a relatively weaker profile correlation to contrast detection, possibly due to the high variability of the contextual modulation profile for inverted faces (see Fig 5A). Lastly, the profile of upright and inverted face contextual modulations was tightly linked (48% of shared variance). The positive correlation between upright and inverted faces both in terms of the profile and the magnitude of contextual modulations yields an unprecedented characterization of their functional overlap at the level of perceptual behavior. It is however important to keep in mind that our profile analyses were conducted on three data points and should therefore be interpreted with caution.

The highly robust contextual modulations for the processing of upright faces is attributed to high level mechanisms implemented in face-specialized visual regions such as the FFA [16, 34, 35]. While some studies have examined the connection between face perception and basic visual abilities like contrast sensitivity, luminance discrimination, and spatial resolution (e.g. [129]), the functional link between face-specific contextual mechanisms and lower-level mechanisms has not been extensively explored. Recent work has however shown that the activity in face-specialized mechanisms is sensitive to face image properties known to be processed in V1 (e.g., orientation, spatial frequency, position). [81] demonstrated that the specialized processing of the upright face preferentially relies on horizontally-structured content (see also: [143–146]). Face processing is also known to depend on the spatial frequency content of the stimulus [13, 78–80, 147–151] and even more so than other object categories ([76, 77], though see [152]).

Past studies reported no correlation, or at best, anecdotal, weak correlation in the magnitude of contextual modulations across tasks irrespective of whether they are measured exclusively within the domain of basic [153–155], or complex stimuli ([156–158]; but see [24]). Considering these past findings, our finding of a correlation in the magnitude of the contextual modulations across visual domains is remarkable. There were several divergences across tasks, among which was the fact that one task required discriminating two subsequently shown eye regions and therefore involved memory while the other did not. Past evidence however shows that the contextual modulations for faces depend on local input strength irrespective of whether a memory component is involved in the task (cf. [14]). The tasks also differed in the nature of the target signal: related to contrast in one task and to shape in the other. Despite these divergences, our design equated the implications of context-target relationships as a function of local input strength across domains, which allowed us to measure contextual modulations on a single, and most importantly, similar metric across tasks. This single metric that is calculated identically across tasks, along with the higher power provided by the Hierarchical Modeling approach may have provided the ideal conditions to observe cross-domain correlation [159–161].

Several studies to date have traced back low and mid-level contextual effects to V1 anatomy. For example, the functionally defined size of V1 has been linked to contextual modulations for basic visual properties such as orientation (tilt illusion: [162, 163]), and even for shape perception (e.g., Ebbinghaus and Ponzo illusions: [164, 165]). Whether similar connections can be established between V1 anatomy and high-level, face contextual modulations is an unanswered question. The absence of a correlation between contrast detection and upright face contextual effects may suggest that there may be no such correlation with V1. Nevertheless, we cannot draw any firm conclusions about the neural workings underlying our findings since the data presented in this study are exclusively behavioral.

In conclusion, by comparing the contextual modulations in a contrast detection task and a facial feature matching task, our study revealed shared variability and functional similarity between low- and high-level contextual mechanisms. Our findings show that low- and high-level, face-specialized contextual modulations have similar working principles while maintaining functional independence. When face-specialized mechanisms are disrupted by inversion, high-level contextual modulations are more functionally connected to low-level mechanisms. The combined study of low- and high-level contextual modulations sheds new light on the functional relationship between the different levels of the visual processing hierarchy, and thus on the functional organization of the visual system.

## Supporting information

S1 File Comparisons to past studies and thresholds across sexes. This file contains extra analyses comparing our results to Goffaux, 2012 and Mannion et al., 2017, along with the comparison of thresholds across sexes and the goodness-of-fit measurements of our model.
(DOCX)

S2 File BRMS model details. We describe the details of the BRMS model we used to obtain thresholds from each subject.
(PDF)

S3 File Psychometric functions of each subject. Psychometric fits of every subject/task/condition acquired from the BRMS model.
(PDF)

S4 File Regression models. Models used for the regression-of-regressions method.
(PDF)

S5 File Model comparison. The details of the model selection procedure is explained in this file.
(PDF)
