# Supplementary material for: Contributions of low- and high-level contextual mechanisms to human face perception
Source: PLoS One. 2023 May 2;18(5):e0285255. doi: 10.1371/journal.pone.0285255 (PMC10153715; doi:10.1371/journal.pone.0285255)
Supplement: S1 File — This file contains extra analyses comparing our results to Goffaux, 2012 and Mannion et al., 2017, along with the comparison of thresholds across sexes and the goodness-of-fit measurements of our model. (DOCX) [file pone.0285255.s001.docx]

# SUPPLEMENTARY

## Eye matching task: Magnitude of contextual modulations.

The eye matching task was a replication of Goffaux (2012). Here, for sake of empirical replication, we used their approach to analyze the data and confirm the quasi-linear decrease of contextual modulation strength as a function of local input dissimilarity. The magnitude of contextual modulations was estimated using the congruency effect; subtracting the proportion of “different” responses in same context/different target trials from different context/same target trials. by subtracting the proportion of “Different” responses in Same context trials from the proportion of “Different” responses in Different context trials. We then calculated the slope of the contextual modulations in terms of increasing dissimilarity for Upright and Inverted faces.

As seen in **Figure S.1,** in Upright faces, the magnitude of contextual modulations decreased as a function of eye region dissimilarity (The slope, *β* = -.0043, 95% CrI [-.005, -.0036], *BF_10_* > 100): the more dissimilar the eyes are, the weaker the contextual modulations. This relative dependence of face contextual modulations on local input strength vanished in Inverted faces (*β* = -.0013, 95% CrI [-.003, .0003], *BF_10_ >* 100). These results replicate the findings of Goffaux (2012), along with the congruency – accuracy scores displayed in Table S.1.

|  | **Upright** |  | | | |
| --- | --- | --- | --- | --- | --- |
|  | 0% | 24% | 36% | 53% | 80% |
| Congruent | .94 (.03) | .6 (.06) | .75 (.05) | .89 (.04) | .97 (.02) |
| Incongruent | .6 (.06) | .19 (.05) | .43 (.06) | .71 (.06) | .93 (.03) |
| Isolated | .9 (.04) | .29 (.05) | .57 (.06) | .83 (.05) | .95 (.03) |
|  | **Inverted** |  | | | |
|  | 0% | 24% | 36% | 53% | 80% |
| Congruent | .87 (.04) | .44 (.06) | .59 (.06) | .80 (.05) | .93 (.03) |
| Incongruent | .77 (.05) | .31 (.06) | .48 (.06) | .74 (.06) | .92 (.03) |
| Isolated | .9 (.04) | .26 (.06) | .44 (.06) | .66 (.06) | .87 (.04) |

*Table S.1.* Congruency scores for the eye matching task. Values display accuracy levels, with standard errors for the respective condition in parentheses.


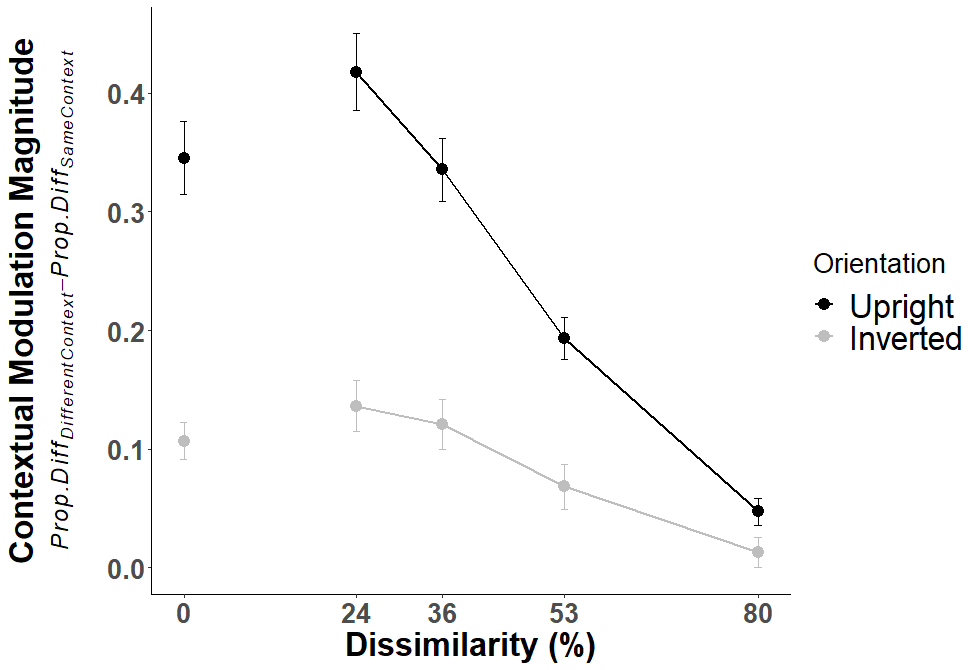

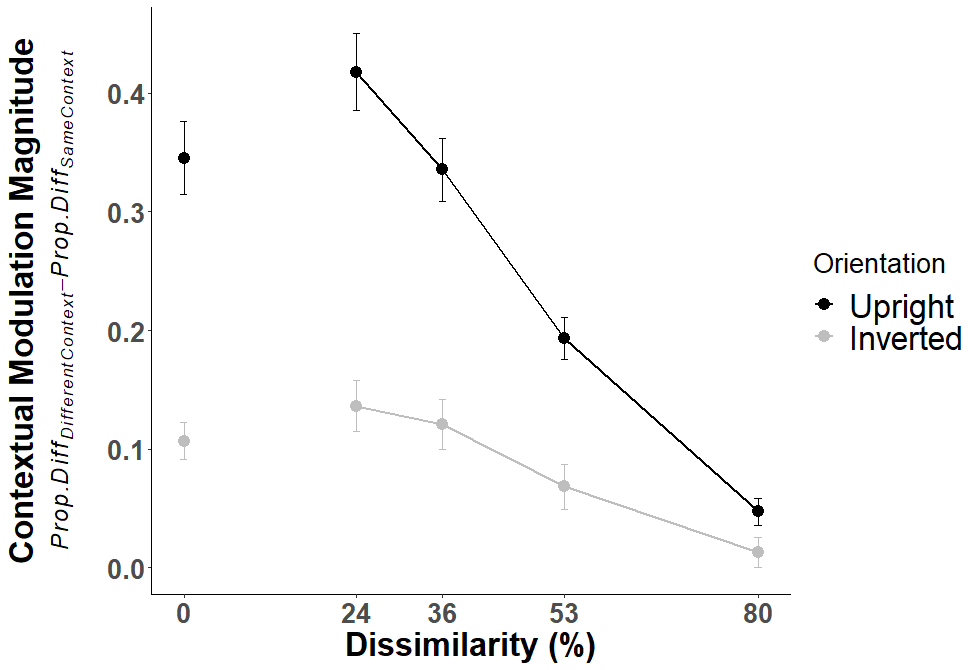


*Figure S.1. Eye matching task.* Contextual modulation magnitude decreases as a function of target feature dissimilarity. The decrease is steeper in Upright compared to Inverted condition.

## Contextual Modulation Magnitude Across Sexes

Despite our sample was not properly balanced to address the effect of gender reliably (47 females, 12 males), we ran Bayesian Independent Sample T-tests on the contextual modulation magnitude values we obtained from the regression of regressions method. We found no difference in any of the three tasks across sexes (see **Figure S.2**). The mean contextual modulation magnitude in Upright Faces were .035 (SE = ±.09) for males and -.009 (±.11) for females (*BF_10_* = 0.32*)*. In Inverted Faces, it was -.014 (±.025) for males and .003 (±.037) for females (*BF_10_* = .32). Lastly, in the Contrast Detection task, mean contextual modulation magnitude was .012 (±.014) for males and -.003 (±.016) for females (*BF_10_* = .34). While these null findings from an unbalanced sample must be interpreted with caution, they agree with past evidence that sex has little or no influence on contextual modulations (Cretenoud et al., 2021; Mannion et al., 2017; Shaqiri et al., 2018).


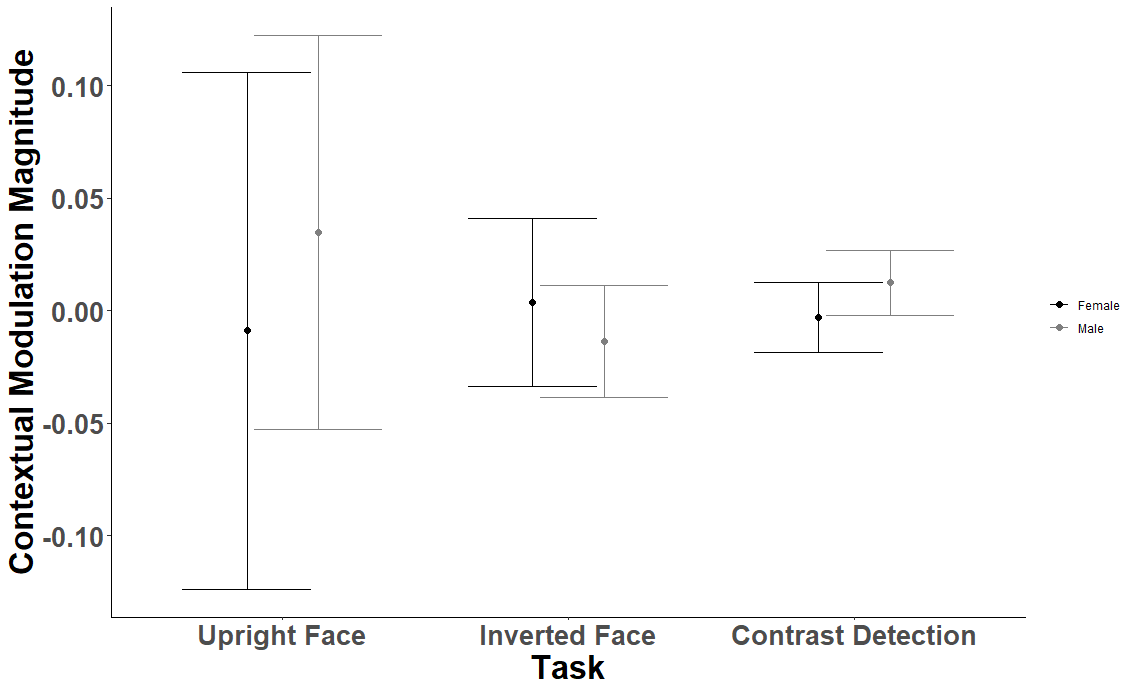

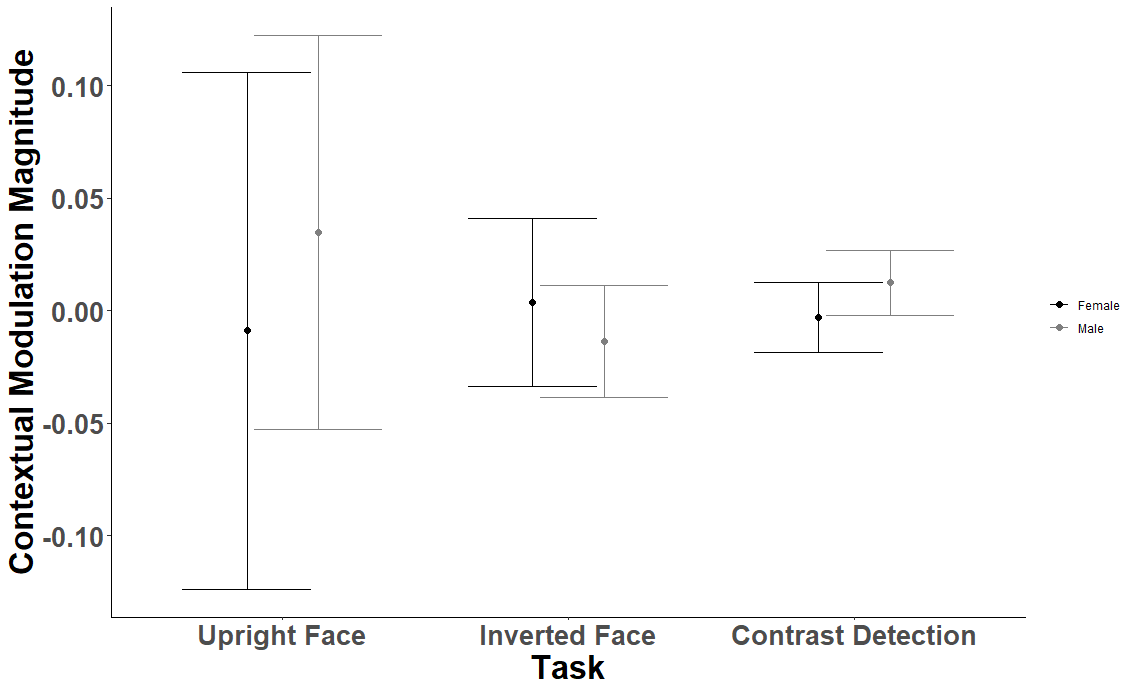


*Figure S.2. Contextual Modulation Magnitude across Sexes.* Error bars display standard errors. Contextual modulation magnitudes showed no difference in any of the tasks between males and females.

## Contrast Detection Task – Comparison with Mannion et al. 2017

In our contrast detection task, we utilized a modified version of the paradigm used in Mannion et al. (2017). We halved the number of response choices compared to Mannion et al. (2017) while keeping the 4 possible target locations. Therefore, chance level differed across studies (50% versus 25%). Despite this difference, we were able to replicate their results. Mean contrast threshold in the Different context condition was 1.37% (95% CI [1.31, 1.43]) in their study whereas it was 1.23% (95% CI [1.12,1.27]) in ours. In Same context condition, mean threshold was 25.49% (95% CI [23.23, 27.71]) in Mannion et al. (2017) whereas it was 18.7% (95% CI [17.6, 19.7]) in ours. The overall pattern of the two contextual conditions were identical.

## Goodness-of-fit measurements for the psychometric functions

We ran deviance tests for the goodness-of-fit of our model as described in Wichmann & Hill (2001). We compared the deviance of the fitted values from the actual data to obtain an empirical deviance value for every single fit (59 subjects, 3 tasks, 3 conditions, 531 fits in total). Fig S.3A shows data from the Contrast Detection task, Same condition data of a single subject, together with the best fitting psychometric function. Fig S.3B shows a histogram of deviance values from 1,000 simulations using the same model’s predictions. The deviance of the empirical data set (D) is 62.675, and the simulated cumulative probability estimate (cpe) is 0.355. The summary statistic deviance, hence, does not indicate a lack of fit. We ran these 1000 model-predicted simulations for each subject/task/condition fit and found that in 88% of the fits, the empirical deviance value were within 95% confidence interval of the simulated deviance values, which points to an overall good fit of our model.


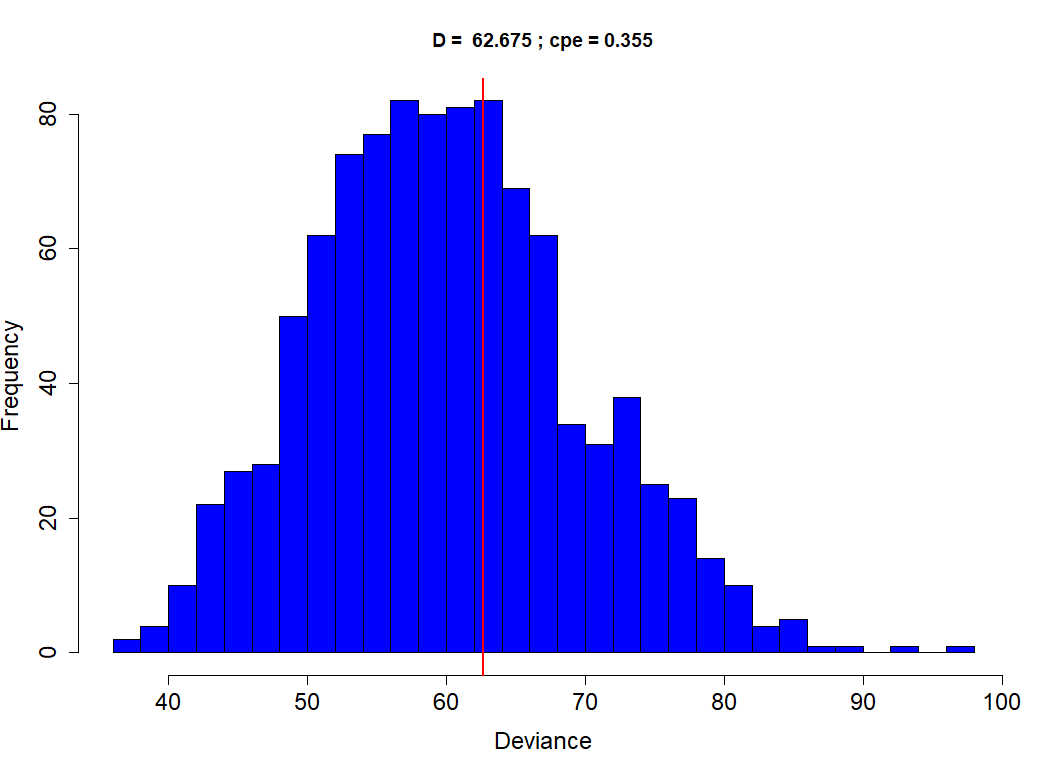


B


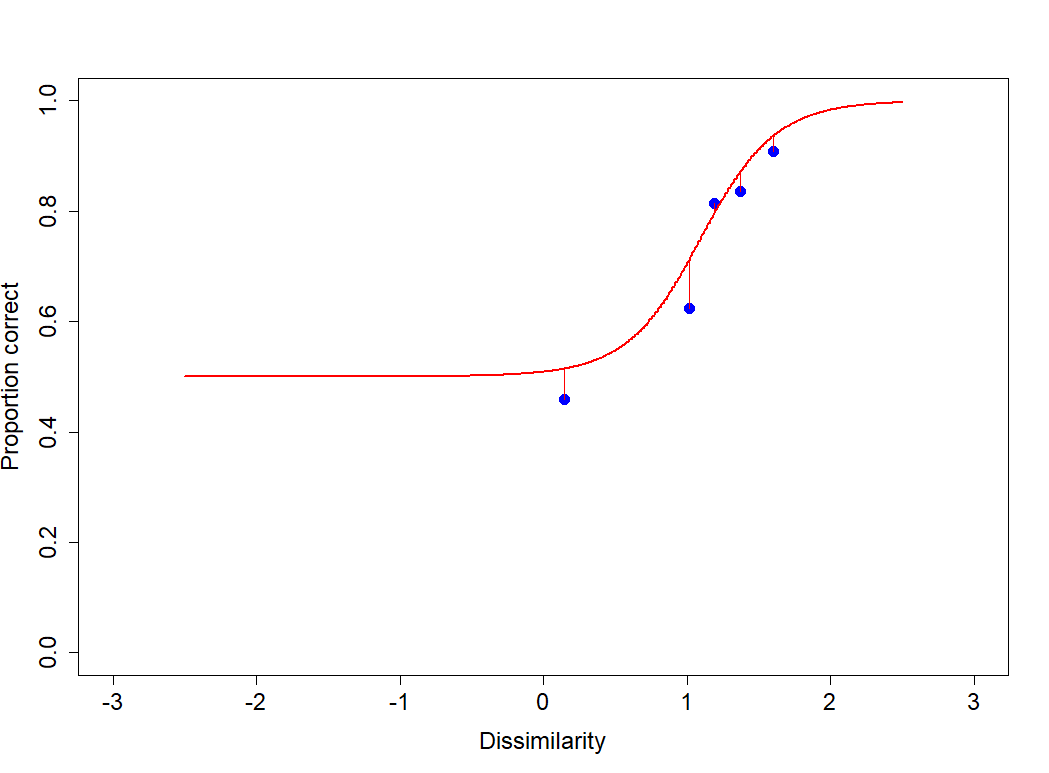


A

*Figure S.3. Goodness-of-fit tests.* **A.** The example data from a single subject with the best-fitting psychometric function. The segments connecting dots to the psychometric function displays the deviation of the model fit from the data. **B.** Histogram of the deviance values of 1,000 simulations using the model predictions. Red line indicates the empirical deviance value obtained from the model fit. Empirical deviance within 95% confidence interval points to a good fit.
