# Supplementary material for: Contributions of low- and high-level contextual mechanisms to human face perception
Source: PLoS One. 2023 May 2;18(5):e0285255. doi: 10.1371/journal.pone.0285255 (PMC10153715; doi:10.1371/journal.pone.0285255)
Supplement: S2 File — We describe the details of the BRMS model we used to obtain thresholds from each subject. (PDF) [file pone.0285255.s002.pdf]

SUPPLEMENTARY: Contributions of low- and high-level contextual mechanisms to human face perception

M. Umut Canoluk[1], Pieter Moors[2], & Valerie Goffaux[1,3,4]

[1] Research Institute for Psychological Science, Université Catholique de Louvain, Louvain-la-Neuve, Belgium
[2] Brain and Cognition, KU Leuven, Leuven, 3000, Belgium
[3] Department of Cognitive Neuroscience, Maastricht University, Maastricht, the Netherlands
[4] Institute of Neuroscience (IoNS), University of Louvain, Louvain-la-Neuve, Belgium

Contents

1 BRMS Model Details **2**

1 BRMS Model Details

```
##  Family: bernoulli
##   Links: mu = identity
## Formula: dv ~ 0.5 * guess + (1 - 0.5 * guess) * inv_logit(eta)
##          eta ~ iv3 * task * cond + (iv3 * task * cond || subject)
##    Data: df_trns (Number of observations: 85467)
##   Draws: 4 chains, each with iter = 6000; warmup = 3000; thin = 1;
##          total post-warmup draws = 12000
##
## Group-Level Effects:
## ~subject (Number of levels: 59)
##                                  Estimate Est.Error l-95% CI u-95% CI Rhat
## sd(eta_Intercept)                    0.98      0.07     0.85     1.13 1.00
## sd(eta_iv3)                          0.70      0.06     0.59     0.83 1.00
## sd(eta_tasklow)                      1.03      0.08     0.89     1.19 1.00
## sd(eta_taskupright)                  0.83      0.07     0.70     0.96 1.00
## sd(eta_condiso)                      0.80      0.06     0.68     0.93 1.00
## sd(eta_condsame)                     0.73      0.06     0.62     0.86 1.00
## sd(eta_iv3:tasklow)                  0.94      0.08     0.80     1.11 1.00
## sd(eta_iv3:taskupright)              0.73      0.06     0.62     0.86 1.00
## sd(eta_iv3:condiso)                  0.69      0.06     0.58     0.82 1.00
## sd(eta_iv3:condsame)                 0.71      0.06     0.60     0.84 1.00
## sd(eta_tasklow:condiso)              0.87      0.08     0.73     1.03 1.00
## sd(eta_taskupright:condiso)          0.83      0.07     0.71     0.98 1.00
## sd(eta_tasklow:condsame)             1.08      0.11     0.89     1.30 1.00
## sd(eta_taskupright:condsame)         0.84      0.07     0.71     1.00 1.00
## sd(eta_iv3:tasklow:condiso)          0.99      0.09     0.82     1.19 1.00
## sd(eta_iv3:taskupright:condiso)      0.78      0.07     0.65     0.92 1.00
## sd(eta_iv3:tasklow:condsame)         0.94      0.09     0.78     1.12 1.00
## sd(eta_iv3:taskupright:condsame)     0.79      0.07     0.66     0.93 1.00
##                                  Bulk_ESS Tail_ESS
## sd(eta_Intercept)                    5573     7463
## sd(eta_iv3)                          9574     9447
## sd(eta_tasklow)                     12304     9267
## sd(eta_taskupright)                 10578     9682
```

```
## sd(eta_condiso)                      10791     9664
## sd(eta_condsame)                     12044     9427
## sd(eta_iv3:tasklow)                  14786     8832
## sd(eta_iv3:taskupright)              13193    10796
## sd(eta_iv3:condiso)                  13834    10232
## sd(eta_iv3:condsame)                 14172     9552
## sd(eta_tasklow:condiso)              15954     8538
## sd(eta_taskupright:condiso)          12955    10176
## sd(eta_tasklow:condsame)             15824     9897
## sd(eta_taskupright:condsame)         14999    10313
## sd(eta_iv3:tasklow:condiso)          17314    10264
## sd(eta_iv3:taskupright:condiso)      15770     9478
## sd(eta_iv3:tasklow:condsame)         15736     9428
## sd(eta_iv3:taskupright:condsame)     15582     9113
##
## Population-Level Effects:
##                               Estimate Est.Error l-95% CI u-95% CI Rhat Bulk_ESS
## eta_Intercept                    -0.14      0.13    -0.40     0.12 1.00     1512
## eta_iv3                           1.60      0.10     1.41     1.79 1.00     2805
## eta_tasklow                       3.80      0.20     3.42     4.19 1.00     5238
## eta_taskupright                   0.88      0.12     0.65     1.12 1.00     3259
## eta_condiso                      -0.96      0.12    -1.19    -0.73 1.00     3827
## eta_condsame                     -0.66      0.11    -0.87    -0.45 1.00     4521
## eta_iv3:tasklow                   3.26      0.25     2.78     3.75 1.00     8638
## eta_iv3:taskupright               0.03      0.11    -0.19     0.25 1.00     5288
## eta_iv3:condiso                   0.24      0.11     0.04     0.45 1.00     6246
## eta_iv3:condsame                  0.29      0.11     0.07     0.51 1.00     5441
## eta_tasklow:condiso               3.50      0.30     2.91     4.09 1.00    10094
## eta_taskupright:condiso          -0.54      0.13    -0.79    -0.28 1.00     7151
## eta_tasklow:condsame             -7.76      0.34    -8.42    -7.09 1.00    15146
## eta_taskupright:condsame         -1.57      0.13    -1.84    -1.31 1.00     6782
## eta_iv3:tasklow:condiso           3.93      0.46     3.05     4.83 1.00    11021
## eta_iv3:taskupright:condiso       0.56      0.14     0.29     0.82 1.00     9391
## eta_iv3:tasklow:condsame         -1.26      0.32    -1.88    -0.65 1.00    12072
## eta_iv3:taskupright:condsame      0.55      0.14     0.28     0.83 1.00     9679
##                               Tail_ESS
```

```
## eta_Intercept                   2924
## eta_iv3                         4857
## eta_tasklow                     7462
## eta_taskupright                 5661
## eta_condiso                     5674
## eta_condsame                    6385
## eta_iv3:tasklow                 9020
## eta_iv3:taskupright             7699
## eta_iv3:condiso                 8336
## eta_iv3:condsame                8228
## eta_tasklow:condiso             9515
## eta_taskupright:condiso         8320
## eta_tasklow:condsame            9930
## eta_taskupright:condsame        8529
## eta_iv3:tasklow:condiso         9063
## eta_iv3:taskupright:condiso     9660
## eta_iv3:tasklow:condsame        9056
## eta_iv3:taskupright:condsame    9715
##
## Draws were sampled using sampling(NUTS). For each parameter, Bulk_ESS
## and Tail_ESS are effective sample size measures, and Rhat is the potential
## scale reduction factor on split chains (at convergence, Rhat = 1).
```
