# Supplementary material for: Contributions of low- and high-level contextual mechanisms to human face perception
Source: PLoS One. 2023 May 2;18(5):e0285255. doi: 10.1371/journal.pone.0285255 (PMC10153715; doi:10.1371/journal.pone.0285255)
Supplement: S3 File — Psychometric fits of every subject/task/condition acquired from the BRMS model. (PDF) [file pone.0285255.s003.pdf]

SUPPLEMENTARY: Contributions of low- and high-level contextual mechanisms to human face perception

M. Umut Canoluk[1], Pieter Moors[2], & Valerie Goffaux[1,3,4]

[1] Research Institute for Psychological Science, Université Catholique de Louvain, Louvain-la-Neuve, Belgium
[2] Brain and Cognition, KU Leuven, Leuven, 3000, Belgium
[3] Department of Cognitive Neuroscience, Maastricht University, Maastricht, the Netherlands
[4] Institute of Neuroscience (IoNS), University of Louvain, Louvain-la-Neuve, Belgium

Contents

1 Psychometric Functions for each subject 2

1 Psychometric Functions for each subject

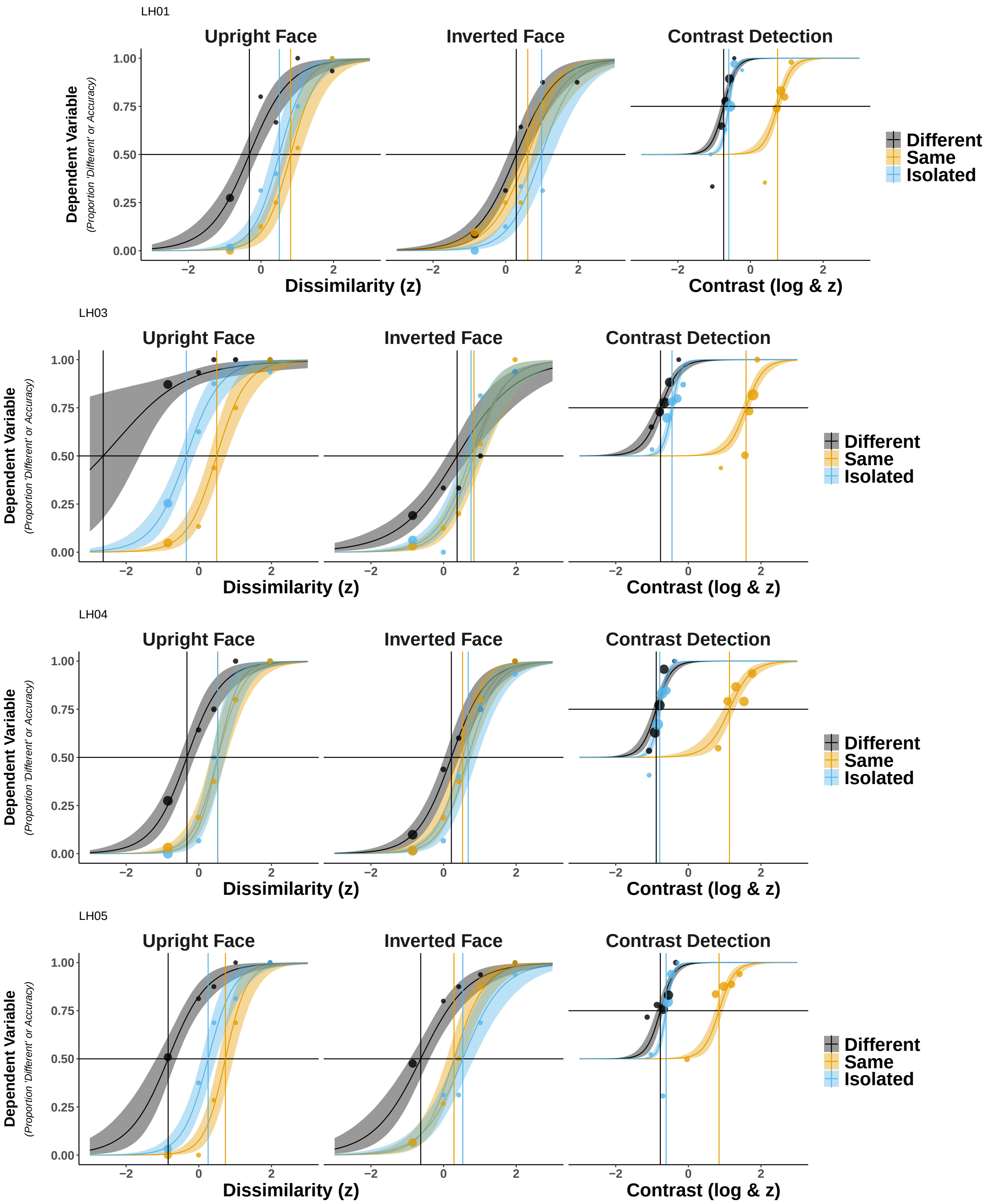

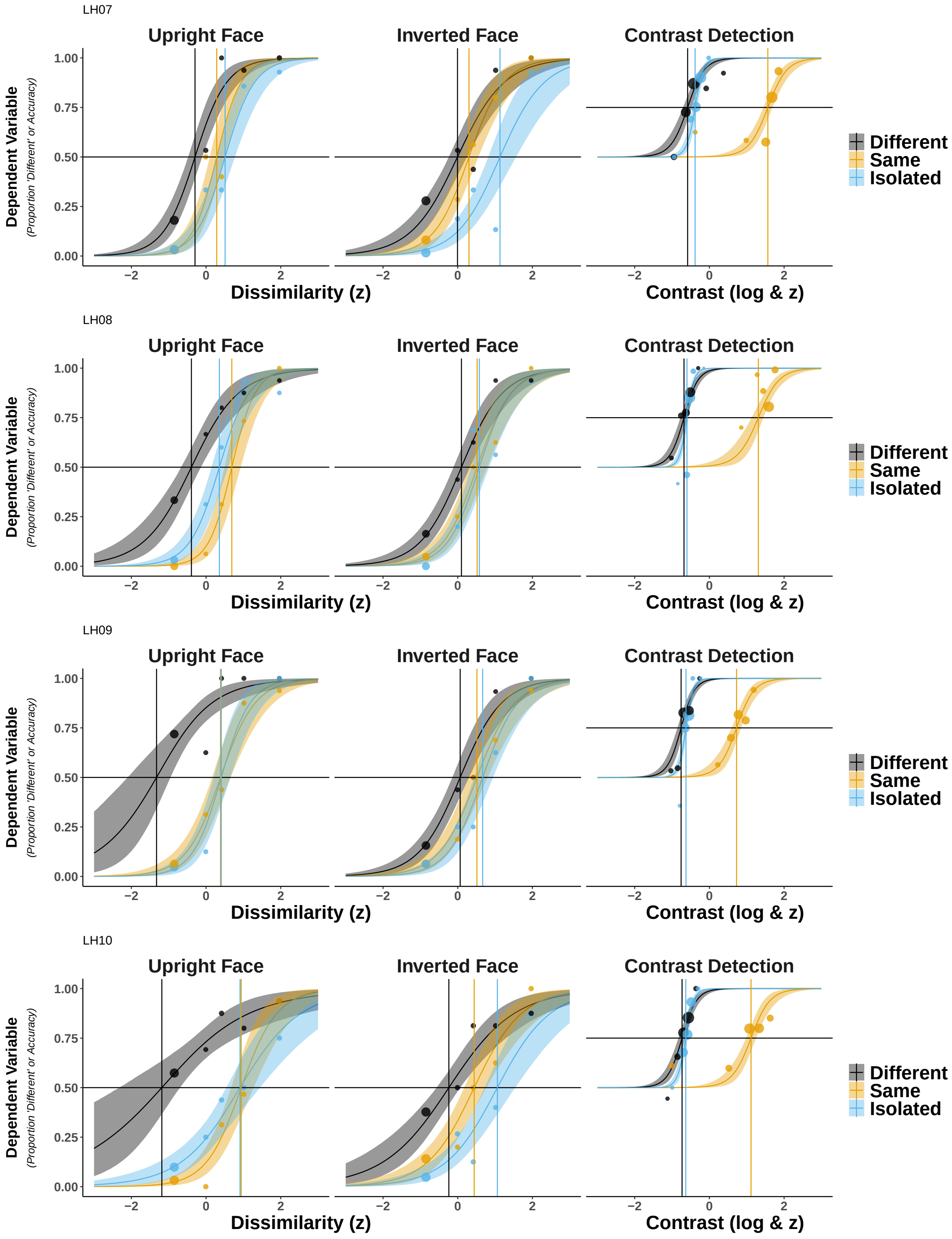
LH07
LH08
LH09
LH10
Upright Face
Inverted Face
Contrast Detection
Dependent Variable
(Proportion 'Different' or Accuracy)
1.00
0.75
0.50
0.25
0.00
−2
0
2
Dissimilarity (z)
Contrast (log & z)
Different
Same
Isolated

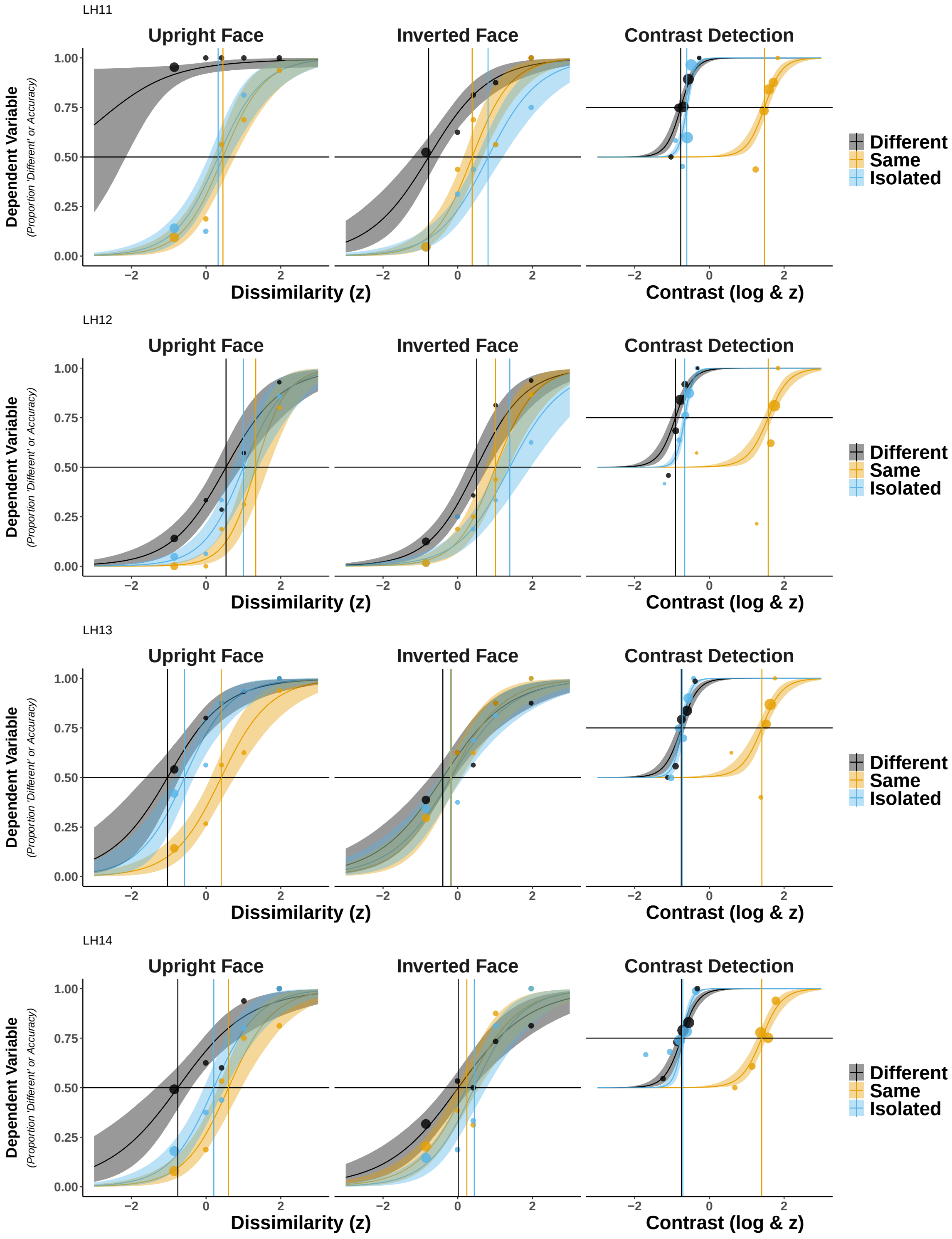
LH11
Upright Face
Inverted Face
Contrast Detection
Dependent Variable
(Proportion 'Different' or Accuracy)
1.00
0.75
0.50
0.25
0.00
−2
0
2
Dissimilarity (z)
Contrast (log & z)
Different
Same
Isolated
LH12
LH13
LH14

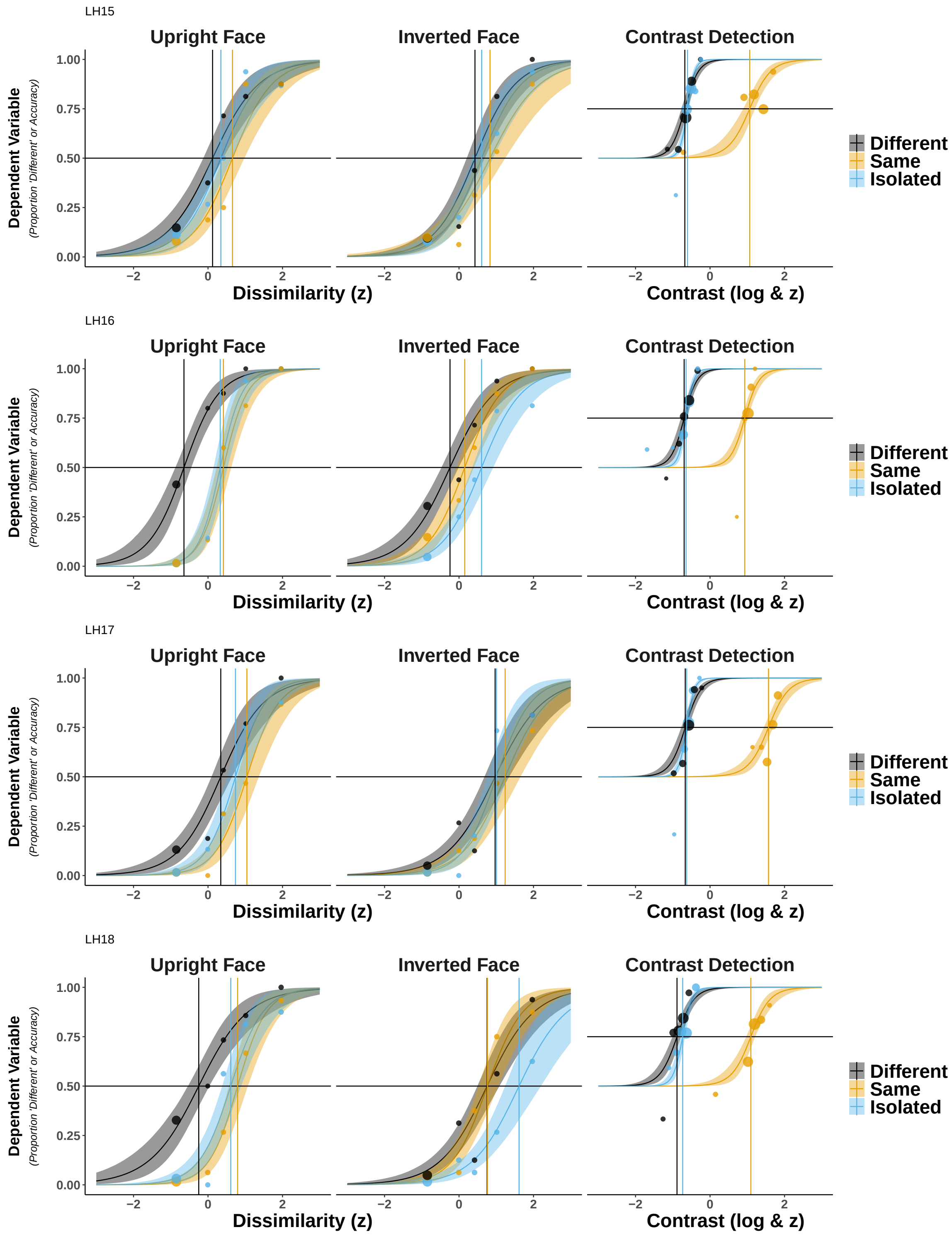
LH15
Upright Face
Inverted Face
Contrast Detection
Dependent Variable
(Proportion 'Different' or Accuracy)
1.00
0.75
0.50
0.25
0.00
−2
0
2
Dissimilarity (z)
Contrast (log & z)
Different
Same
Isolated
LH16
LH17
LH18

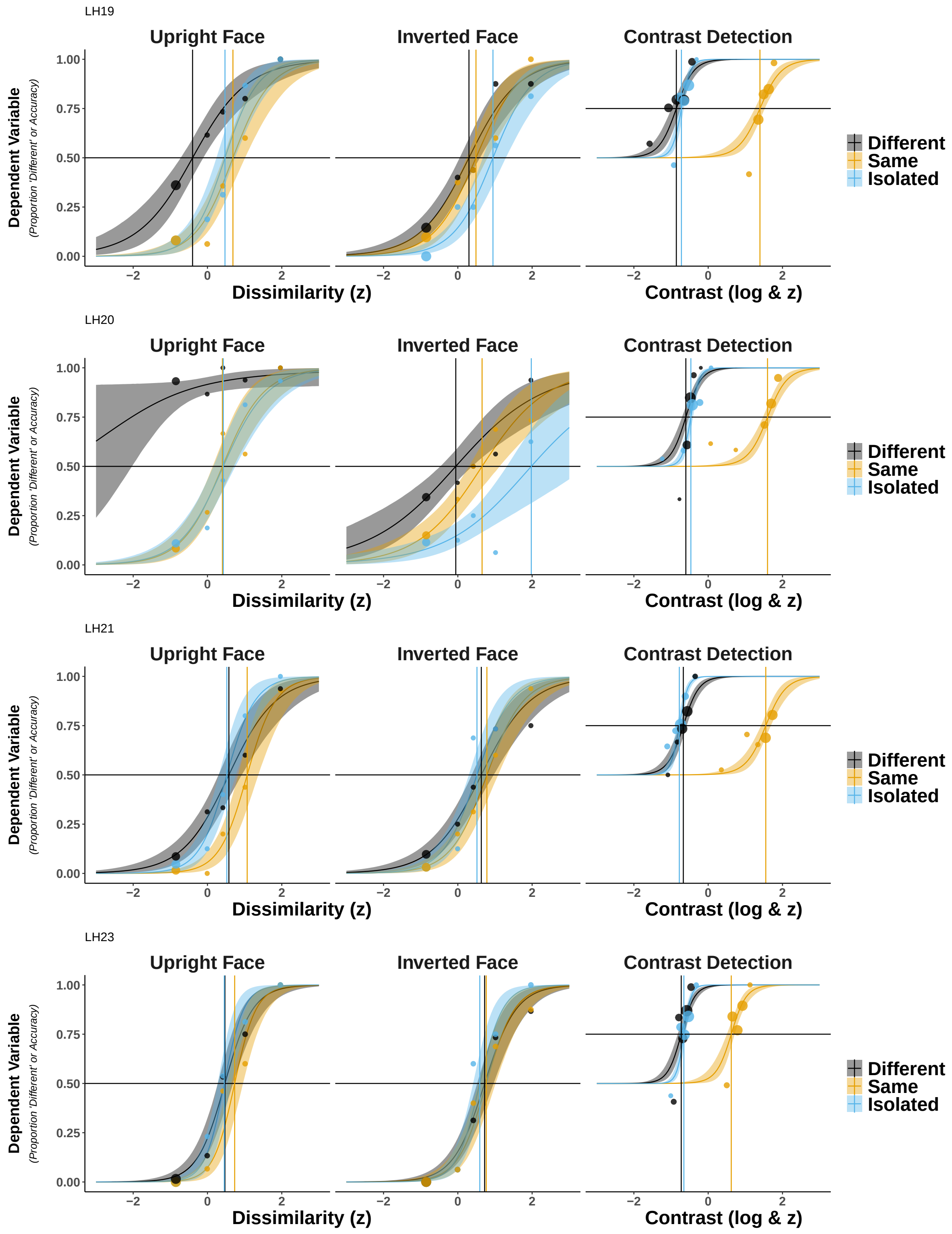
LH19
LH20
LH21
LH23
Upright Face
Inverted Face
Contrast Detection
Dependent Variable
(Proportion 'Different' or Accuracy)
Dissimilarity (z)
Contrast (log & z)
Different
Same
Isolated

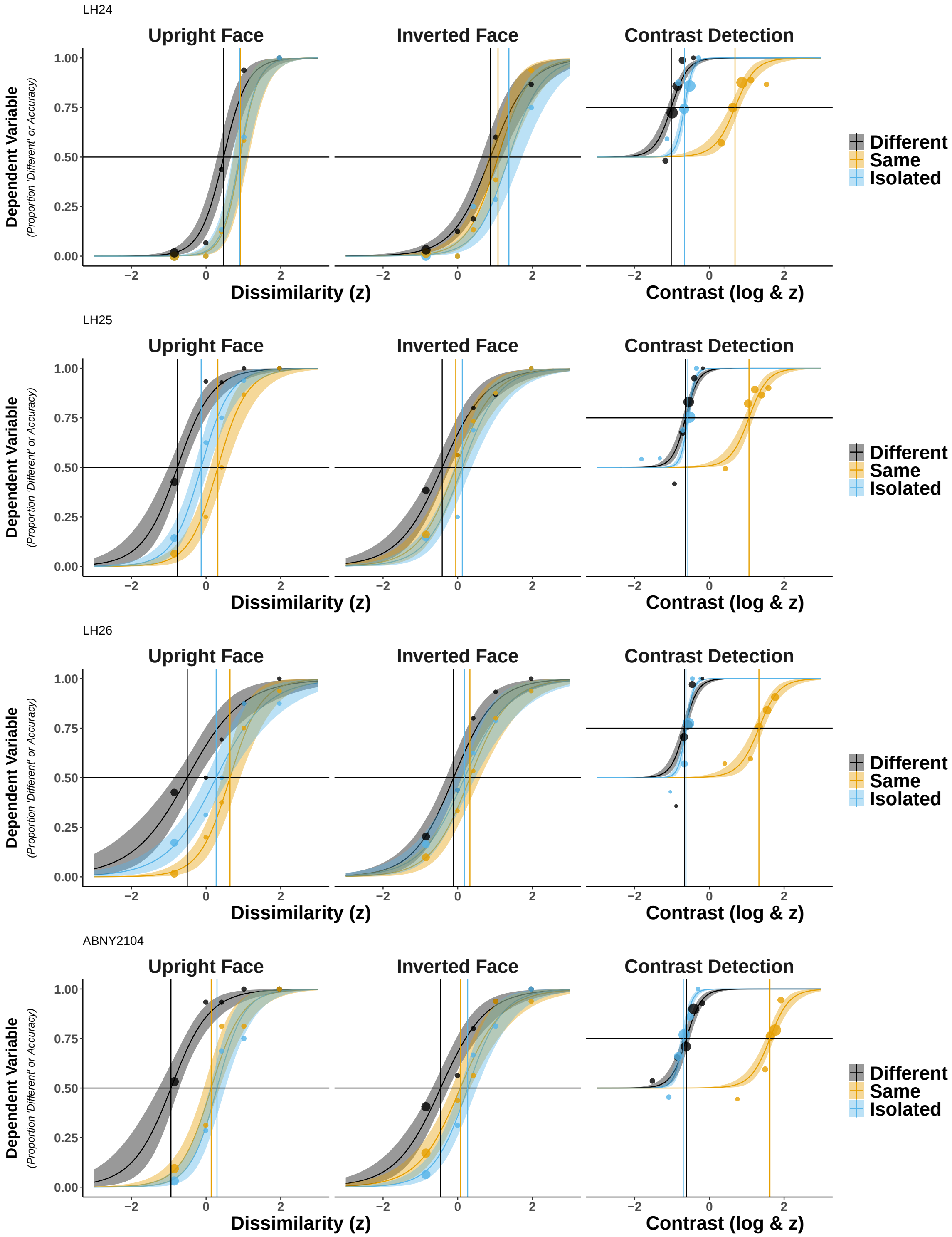
LH24
LH25
LH26
ABNY2104
Upright Face
Inverted Face
Contrast Detection
Dependent Variable
(Proportion 'Different' or Accuracy)
Dissimilarity (z)
Contrast (log & z)
Different
Same
Isolated

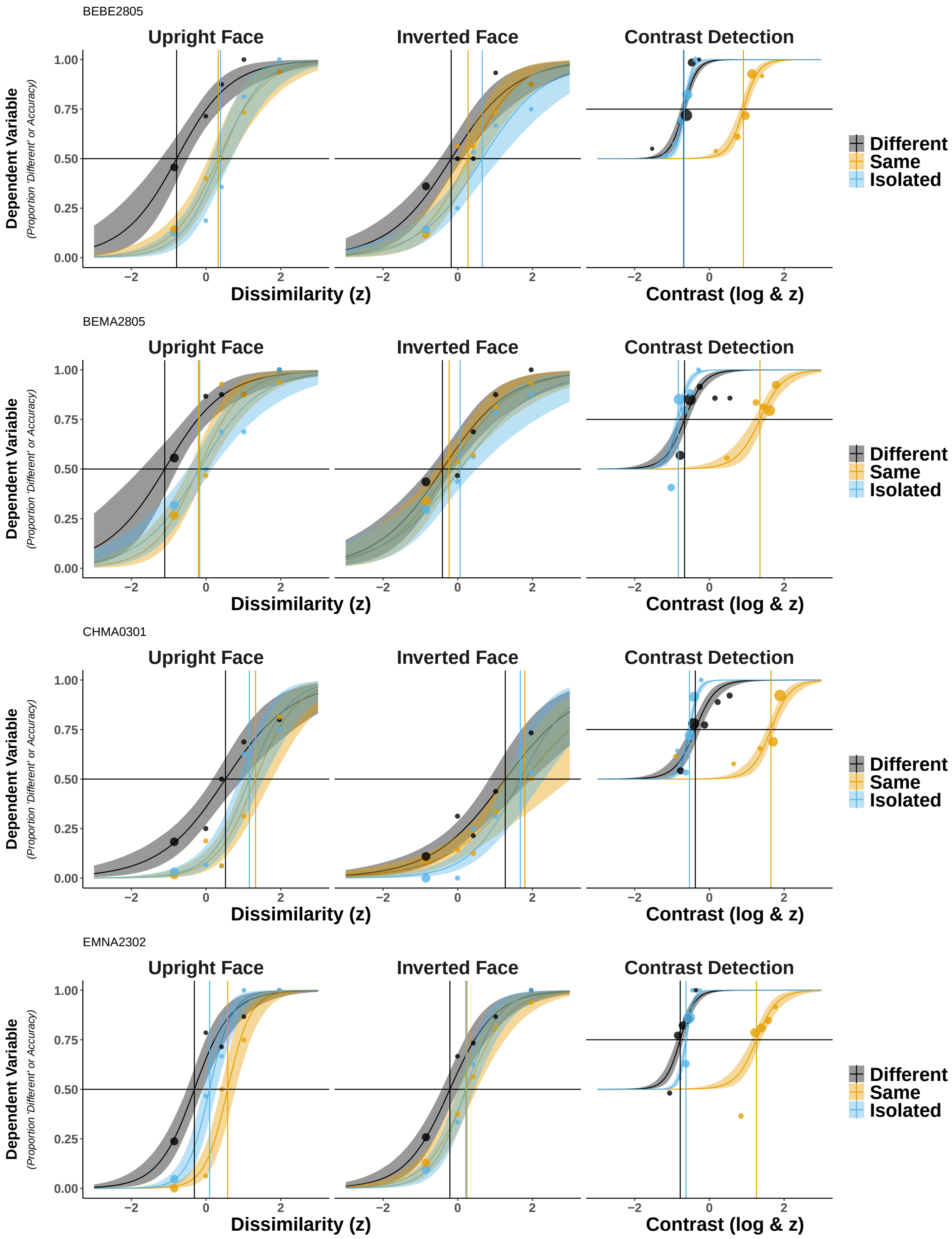
BEBE2805
Upright Face
Inverted Face
Contrast Detection
Dependent Variable
(Proportion 'Different' or Accuracy)
1.00
0.75
0.50
0.25
0.00
−2
0
2
Dissimilarity (z)
Contrast (log & z)
Different
Same
Isolated
BEMA2805
Upright Face
Inverted Face
Contrast Detection
Dependent Variable
(Proportion 'Different' or Accuracy)
Dissimilarity (z)
Contrast (log & z)
Different
Same
Isolated
CHMA0301
Upright Face
Inverted Face
Contrast Detection
Dependent Variable
(Proportion 'Different' or Accuracy)
Dissimilarity (z)
Contrast (log & z)
Different
Same
Isolated
EMNA2302
Upright Face
Inverted Face
Contrast Detection
Dependent Variable
(Proportion 'Different' or Accuracy)
Dissimilarity (z)
Contrast (log & z)
Different
Same
Isolated

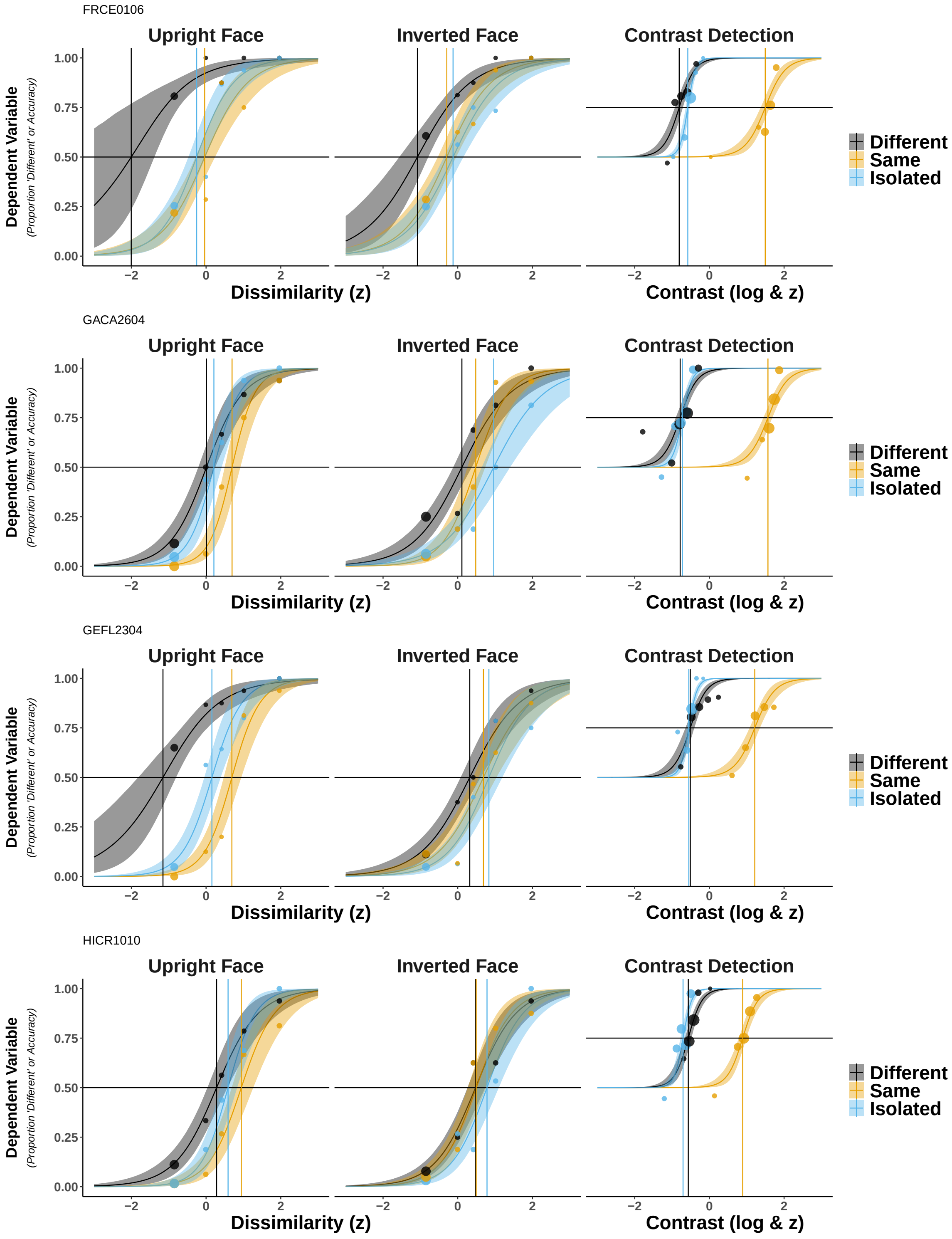
FRCE0106
GACA2604
GEFL2304
HICR1010
Upright Face
Inverted Face
Contrast Detection
Dependent Variable
(Proportion 'Different' or Accuracy)
Dissimilarity (z)
Contrast (log & z)
Different
Same
Isolated

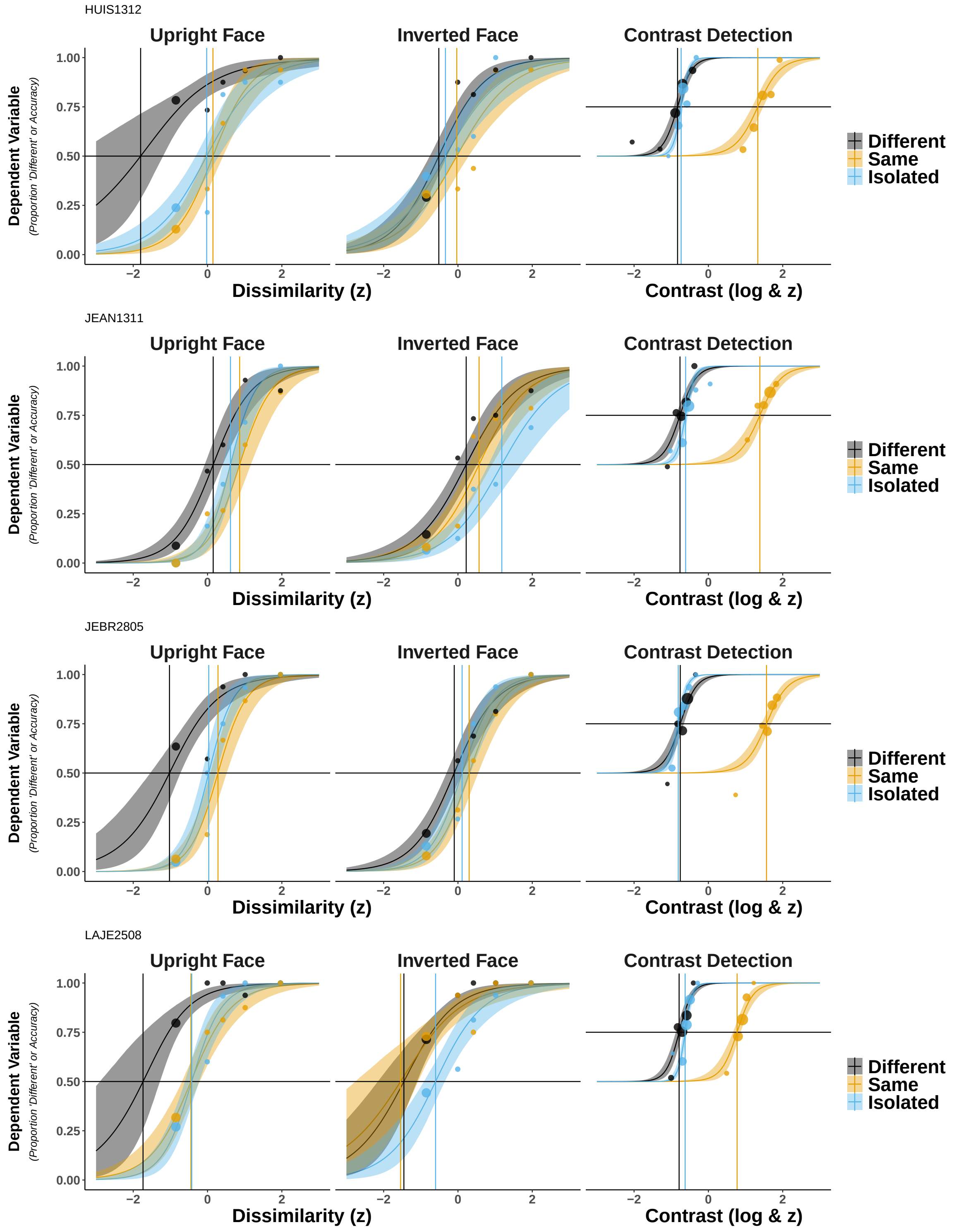
HUIS1312
Upright Face
Inverted Face
Contrast Detection
Dependent Variable
(Proportion 'Different' or Accuracy)
1.00
0.75
0.50
0.25
0.00
−2
0
2
Dissimilarity (z)
Contrast (log & z)
Different
Same
Isolated
JEAN1311
JEBR2805
LAJE2508

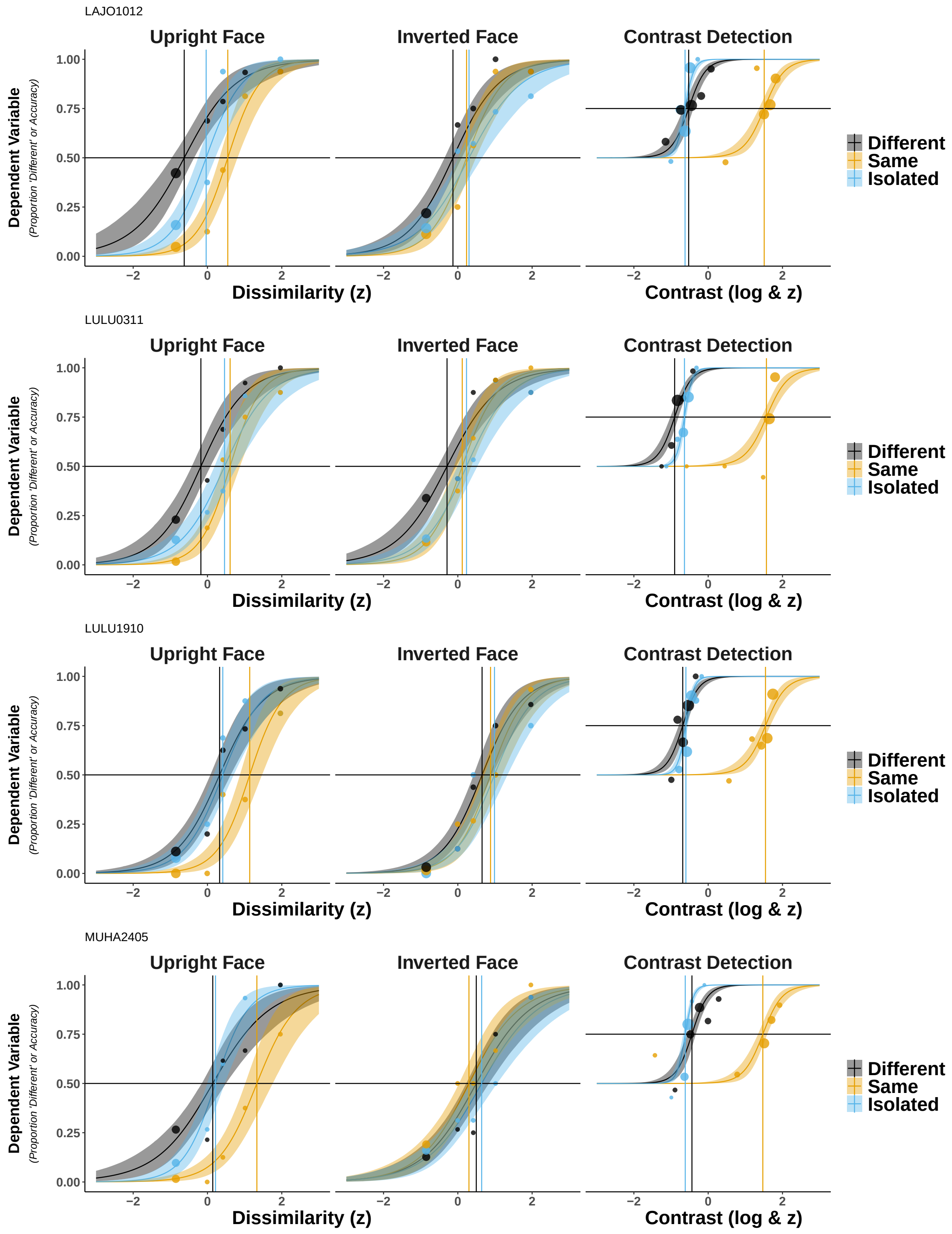
LAJO1012
Upright Face
Inverted Face
Contrast Detection
Dependent Variable
(Proportion 'Different' or Accuracy)
1.00
0.75
0.50
0.25
0.00
−2
0
2
Dissimilarity (z)
Contrast (log & z)
Different
Same
Isolated
LULU0311
LULU1910
MUHA2405

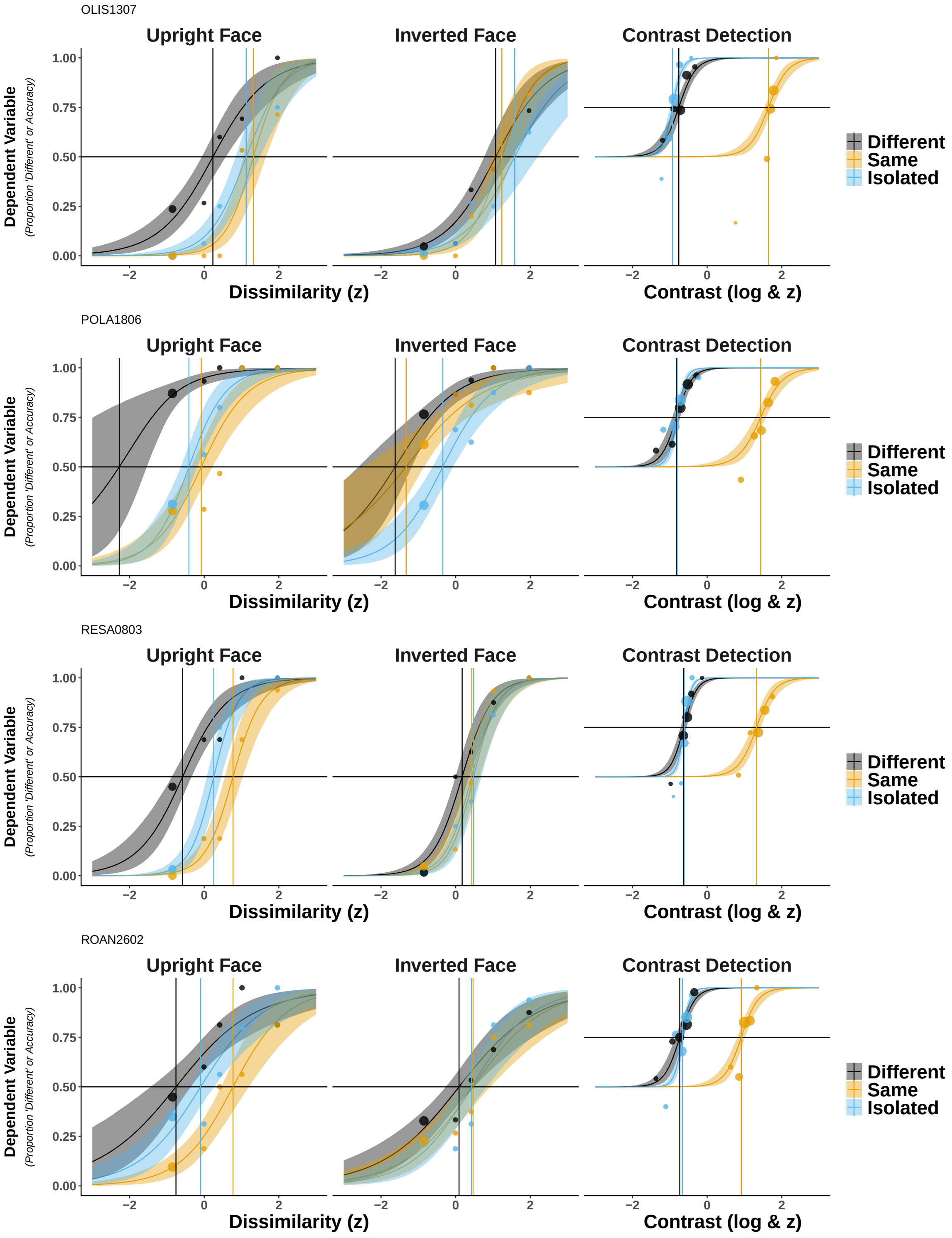

OLIS1307
POLA1806
RESA0803
ROAN2602
Upright Face
Inverted Face
Contrast Detection
Dependent Variable
(Proportion 'Different' or Accuracy)
Dissimilarity (z)
Contrast (log & z)
Different
Same
Isolated

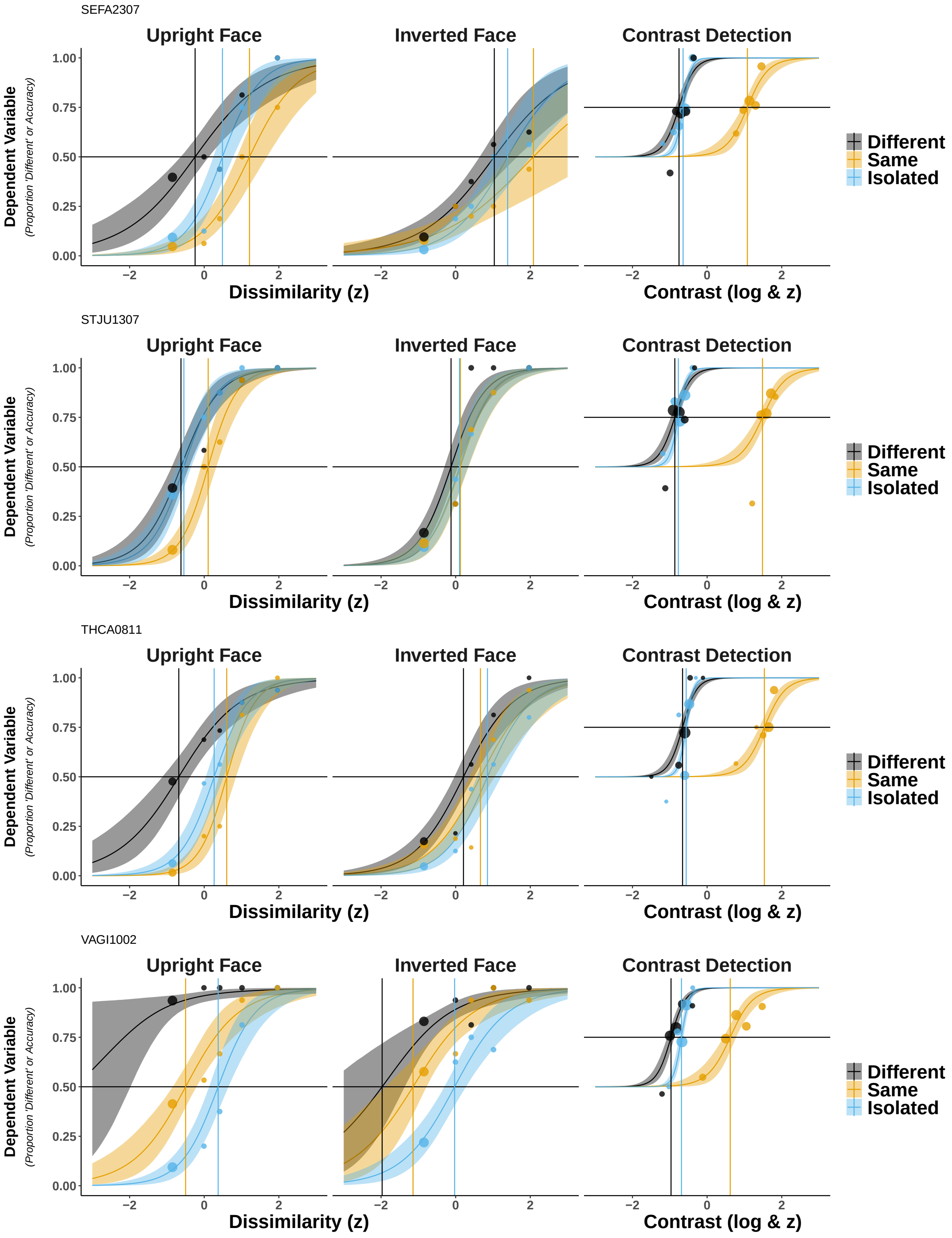
SEFA2307
Upright Face
Inverted Face
Contrast Detection
Dependent Variable
(Proportion 'Different' or Accuracy)
Dissimilarity (z)
Contrast (log & z)
Different
Same
Isolated
STJU1307
THCA0811
VAGI1002

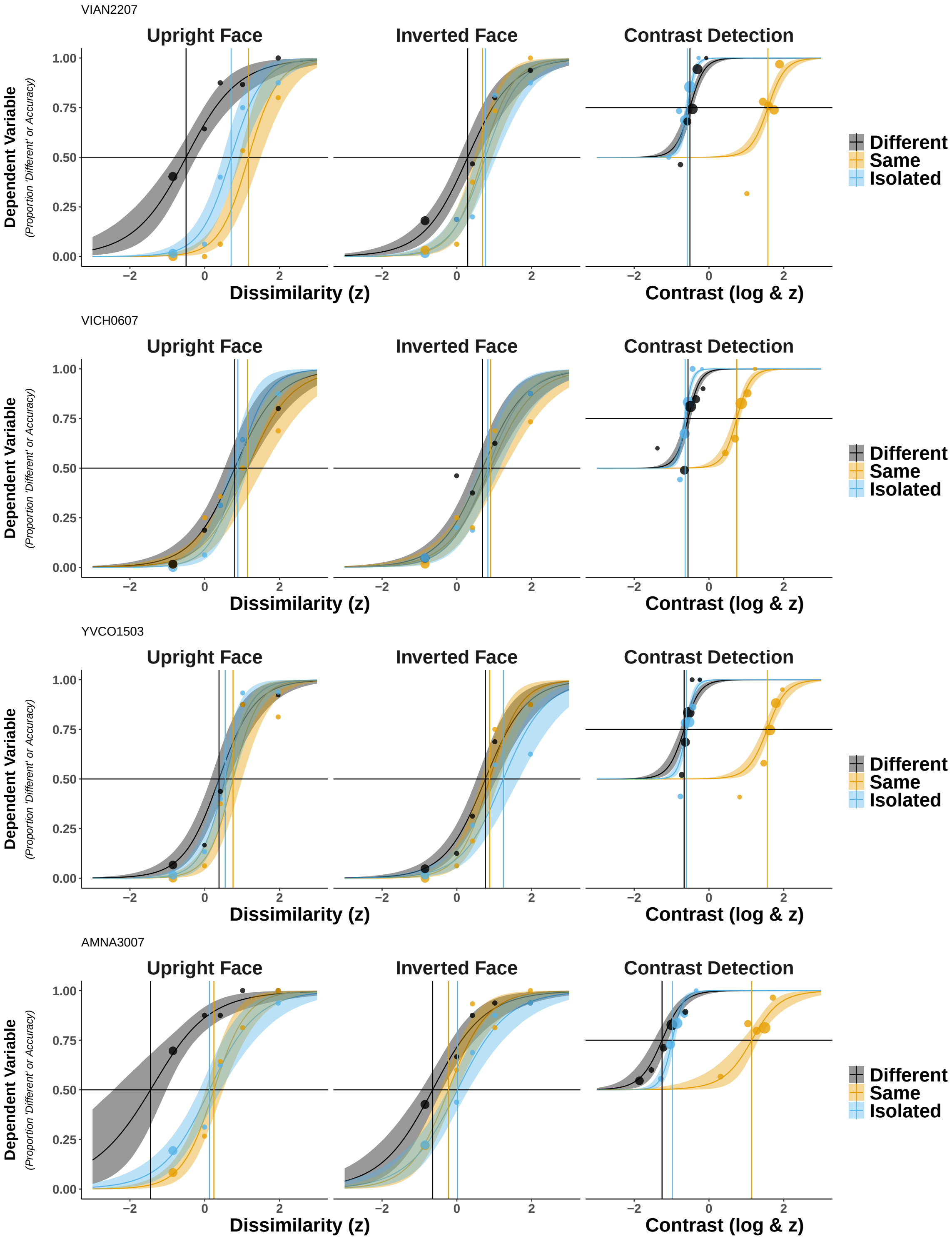
VIAN2207
VICH0607
YVCO1503
AMNA3007
Upright Face
Inverted Face
Contrast Detection
Dependent Variable
(Proportion 'Different' or Accuracy)
1.00
0.75
0.50
0.25
0.00
−2
0
2
Dissimilarity (z)
Contrast (log & z)
Different
Same
Isolated

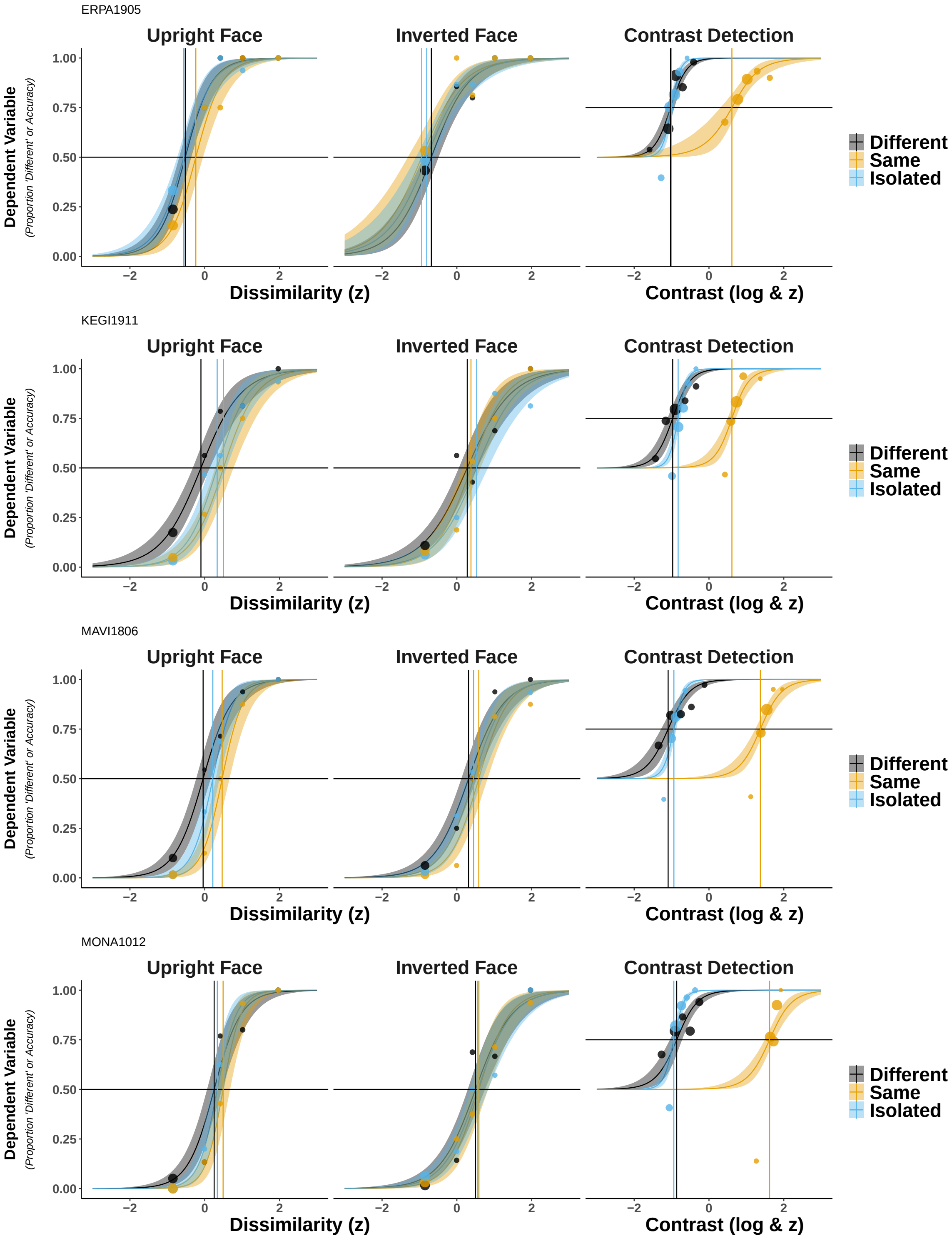
ERPA1905
KEGI1911
MAVI1806
MONA1012
Upright Face
Inverted Face
Contrast Detection
Dependent Variable
(Proportion 'Different' or Accuracy)
1.00
0.75
0.50
0.25
0.00
−2
0
2
Dissimilarity (z)
Contrast (log & z)
Different
Same
Isolated

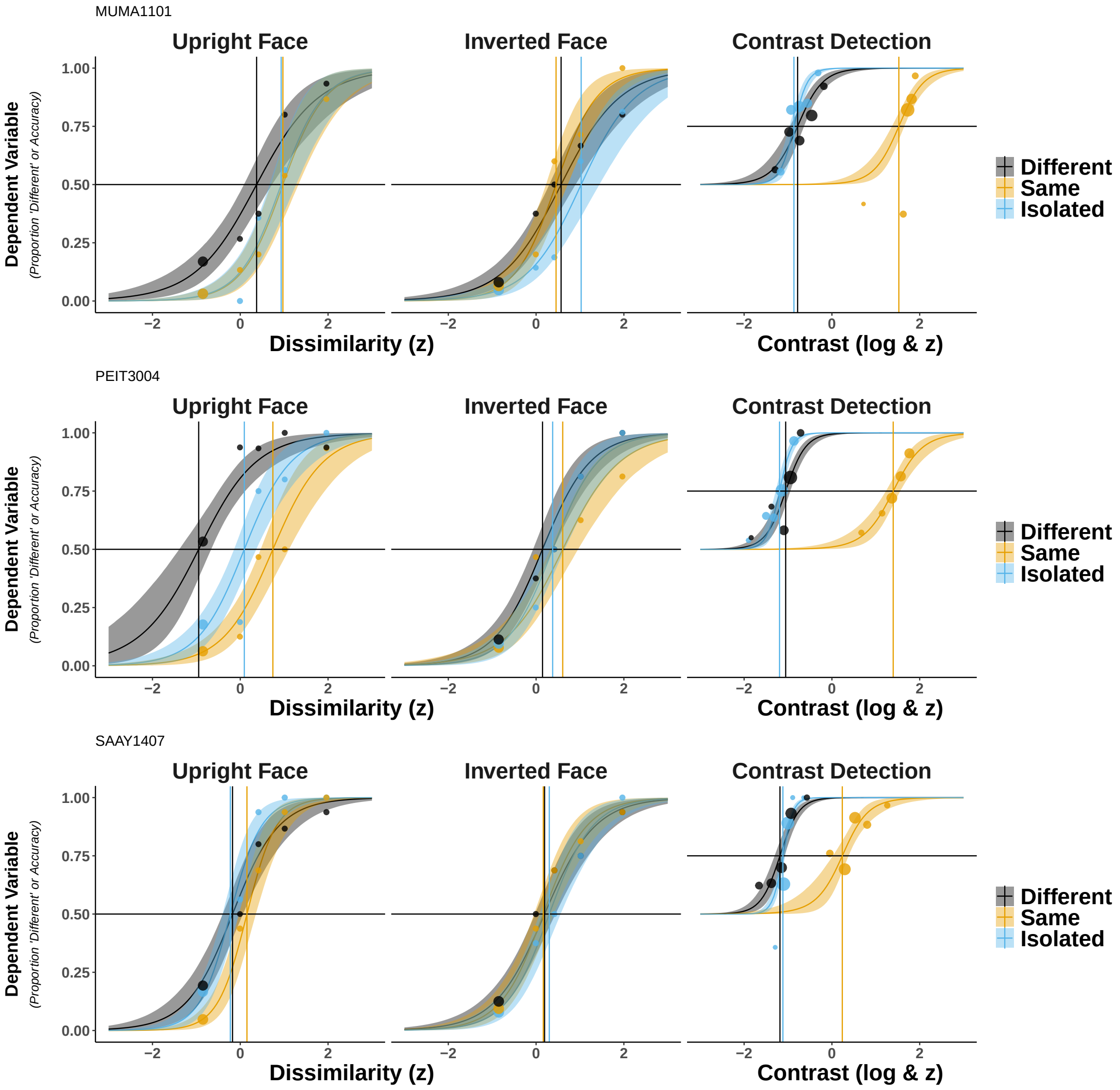
MUMA1101
PEIT3004
SAAY1407
Upright Face
Inverted Face
Contrast Detection
Dependent Variable
(Proportion 'Different' or Accuracy)
Dissimilarity (z)
Contrast (log & z)
Different
Same
Isolated
