# Supplementary material for: Contributions of low- and high-level contextual mechanisms to human face perception
Source: PLoS One. 2023 May 2;18(5):e0285255. doi: 10.1371/journal.pone.0285255 (PMC10153715; doi:10.1371/journal.pone.0285255)

SUPPLEMENTARY 3: Contributions of low- and high-level contextual mechanisms to human face perception

M. Umut Canoluk[1], Pieter Moors[2], & Valerie Goffaux[1,3,4]

[1] Research Institute for Psychological Science, Université Catholique de Louvain, Louvain-la-Neuve, Belgium
[2] Brain and Cognition, KU Leuven, Leuven, 3000, Belgium
[3] Department of Cognitive Neuroscience, Maastricht University, Maastricht, the Netherlands
[4] Institute of Neuroscience (IoNS), University of Louvain, Louvain-la-Neuve, Belgium

The straight lines from the points to the regression line display the residuals for a given data point

First level Regressions

Upright - Isolated ~ Different.

```
##
## Call:
## lm(formula = up_diff ~ up_iso, data = b2)
##
## Coefficients:
## (Intercept)       up_iso
##     -0.9717       1.1103
```

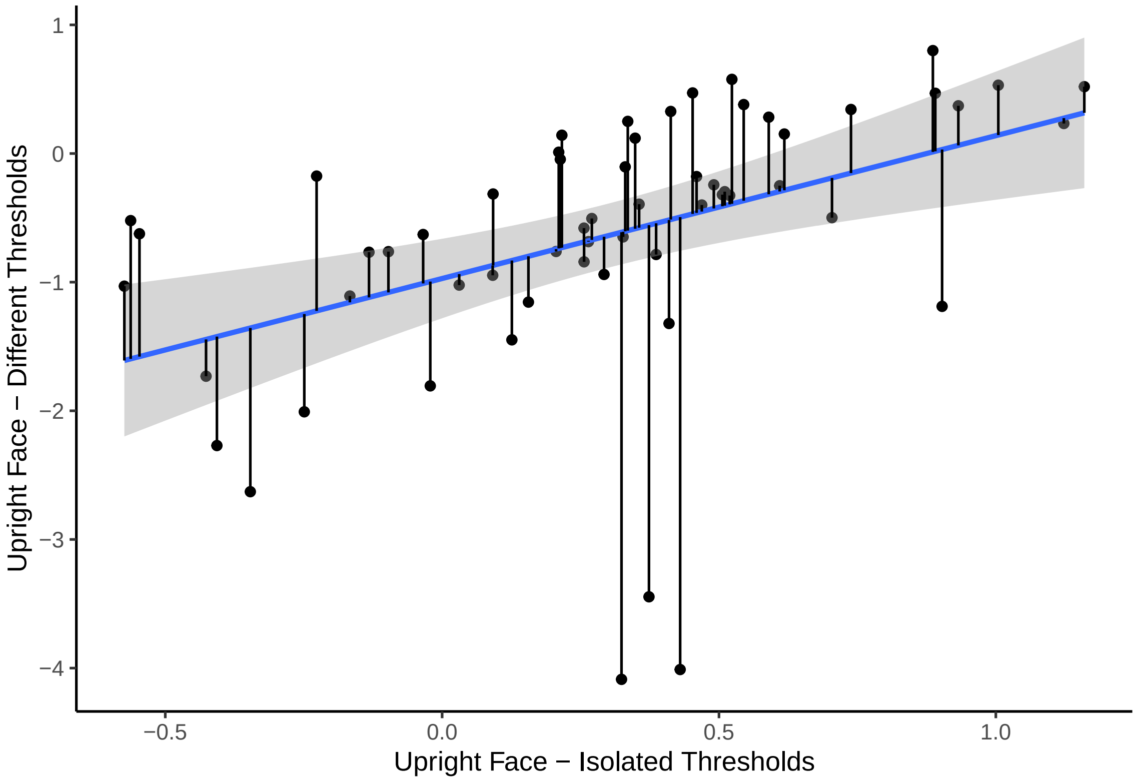

Upright - Isolated ~ Same.

```
##
## Call:
## lm(formula = up_same ~ up_iso, data = b2)
##
## Coefficients:
## (Intercept)       up_iso
##      0.3653       0.7565
```

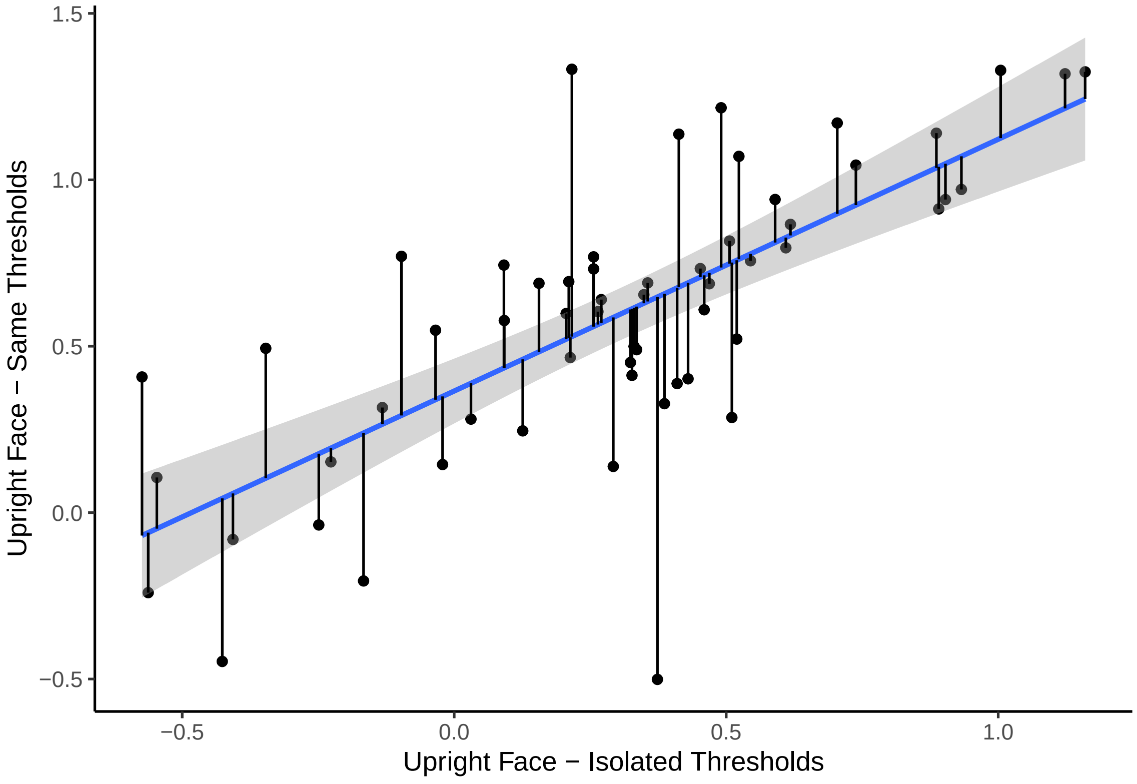

Inverted - Isolated ~ Different.

```
##
## Call:
## lm(formula = inv_diff ~ inv_iso, data = b2)
##
## Coefficients:
## (Intercept)      inv_iso
##     -0.4474       0.8263
```

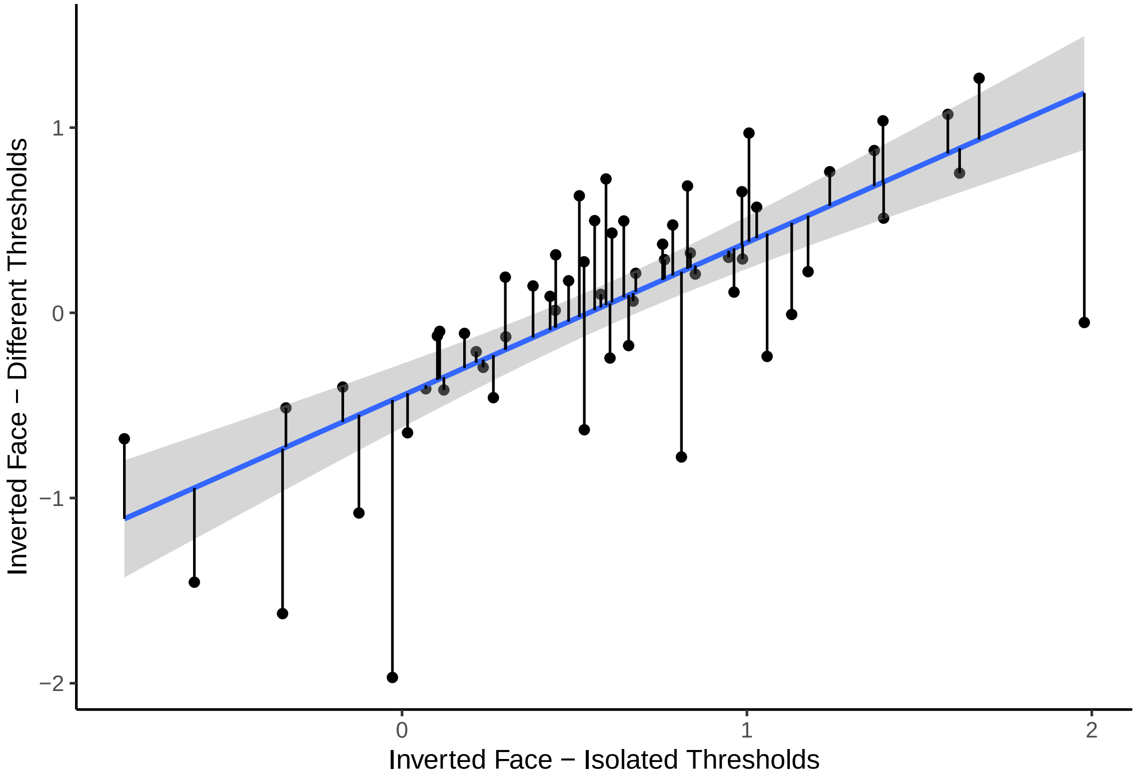

Inverted - Isolated ~ Same.

```
##
## Call:
## lm(formula = inv_same ~ inv_iso, data = b2)
##
## Coefficients:
## (Intercept)      inv_iso
##     -0.1502       0.8981
```

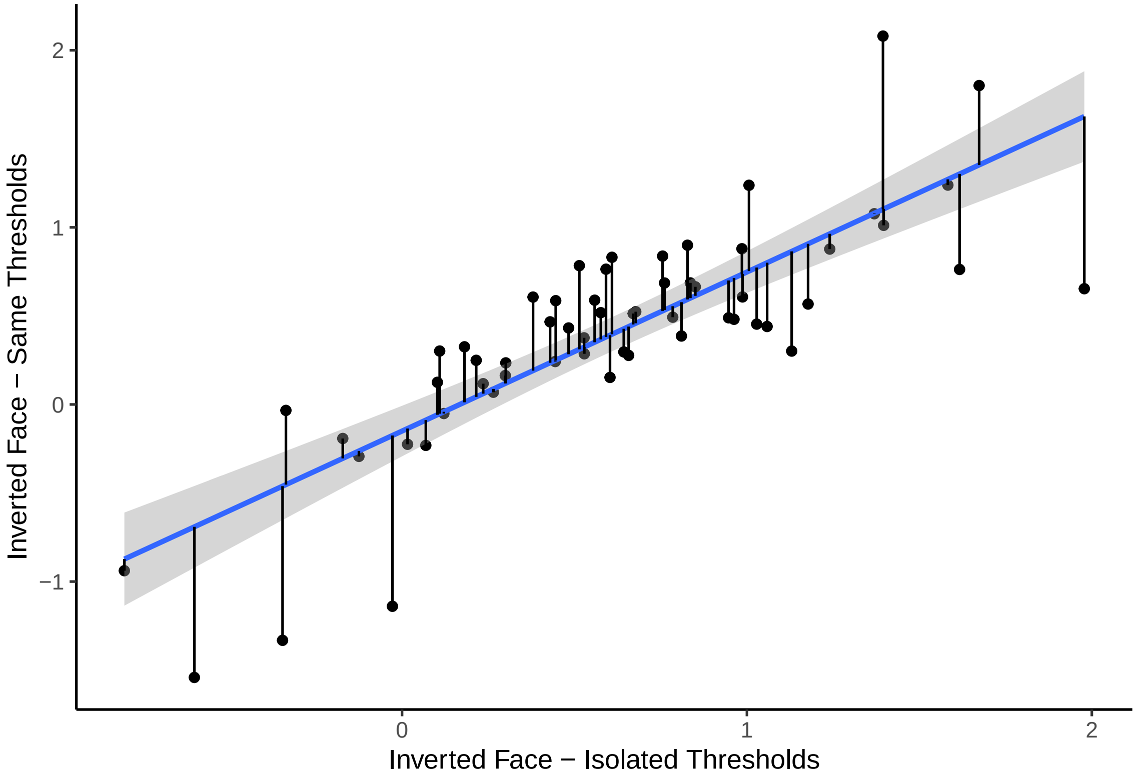

Contrast Detection - Isolated ~ Different.

```
##
## Call:
## lm(formula = low_diff ~ low_iso, data = b2)
##
## Coefficients:
## (Intercept)      low_iso
##     -0.2243       0.7700
```

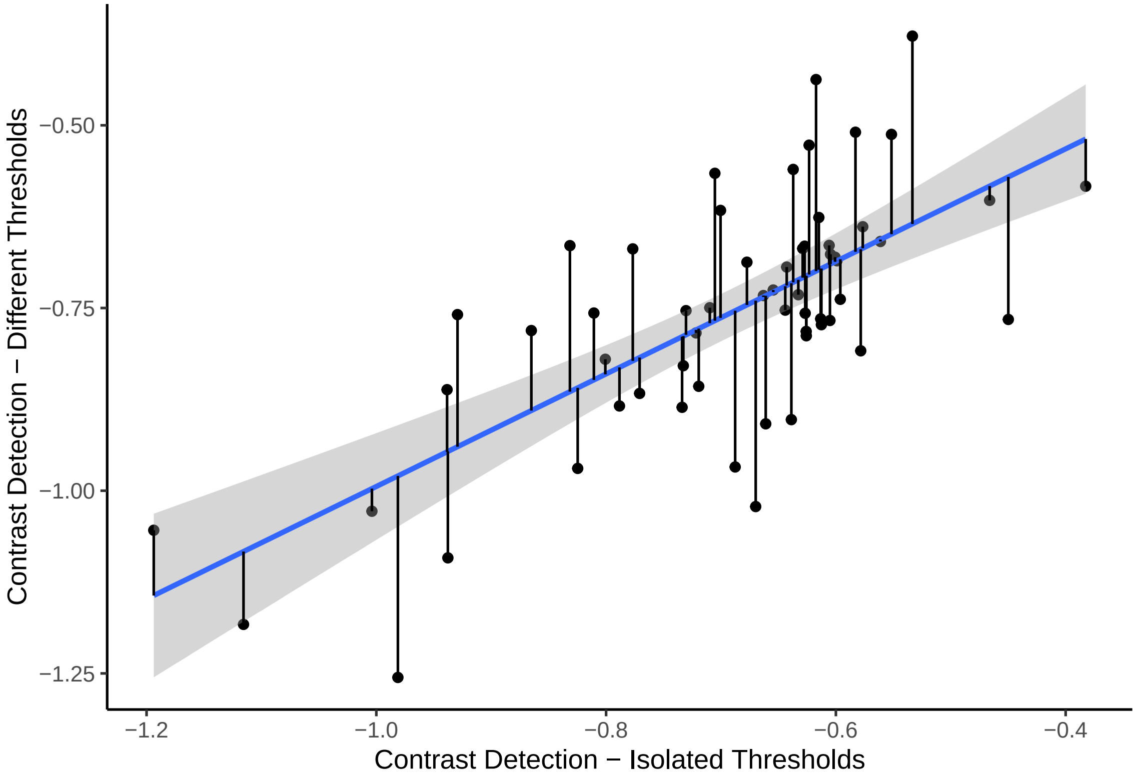

Contrast Detection - Isolated ~ Same.

```
##
## Call:
## lm(formula = low_same ~ low_iso, data = b2)
##
## Coefficients:
## (Intercept)      low_iso
##      1.5485       0.4385
```

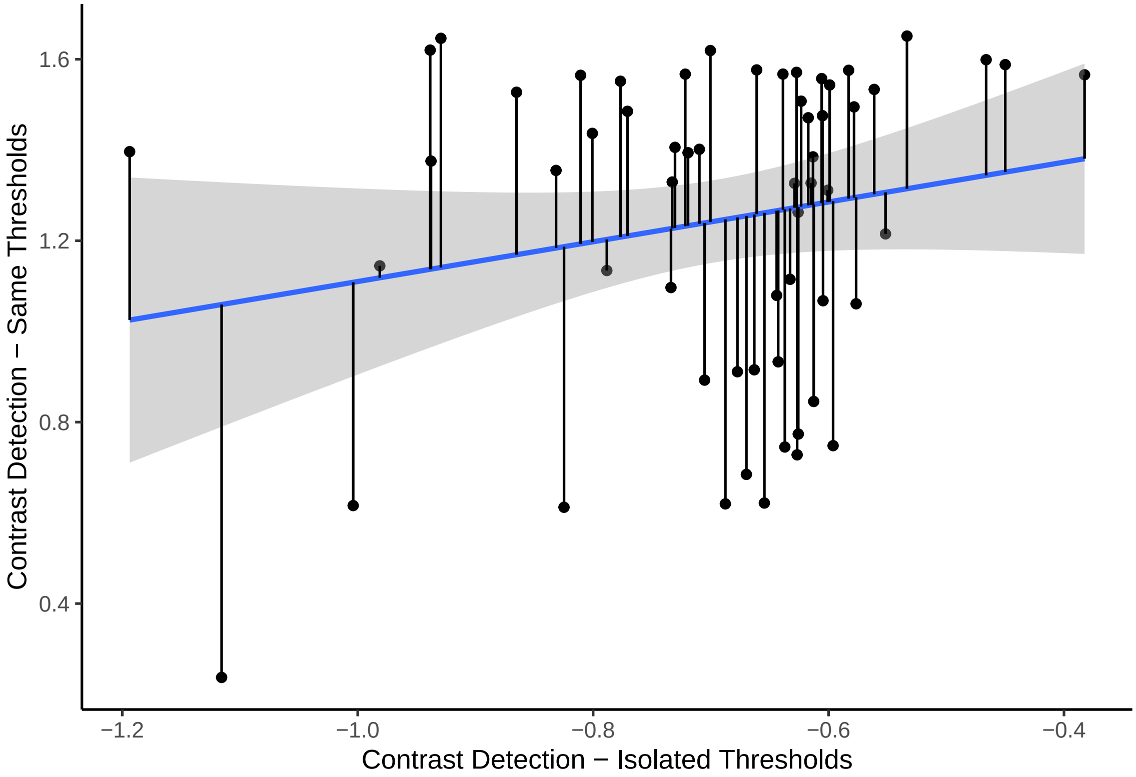

Second Level Regressions

The residuals obtained from the first level regressions are utilized in the second level regressions.

The residuals of these second-level regressions are used as the **magnitude of contextual modulations** for a given task.

Upright Face - Different Residuals ~ Same Residuals.

```
##
## Call:
## lm(formula = uidresid ~ uisresid, data = b3)
##
## Coefficients:
## (Intercept)     uisresid
##   4.915e-17    1.467e+00
```

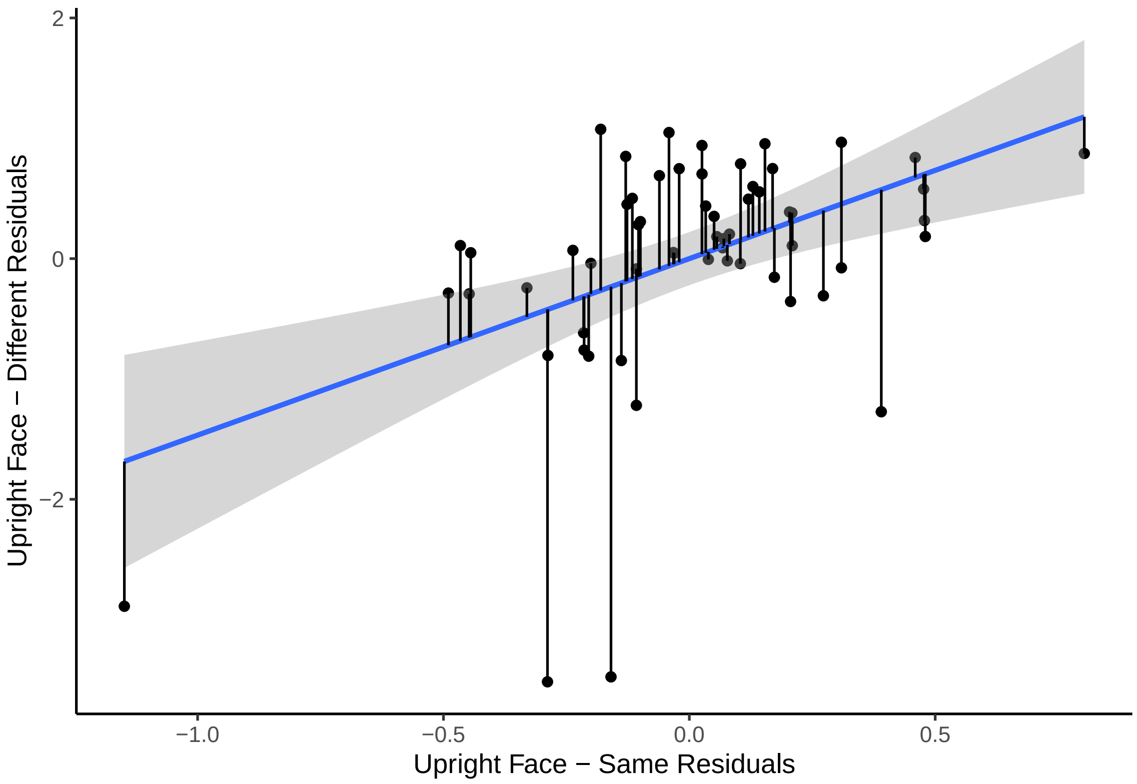

Inverted Face - Different Residuals ~ Same Residuals.

```
##
## Call:
## lm(formula = iidresid ~ iisresid, data = b3)
##
## Coefficients:
## (Intercept)     iisresid
##   3.867e-18    9.511e-01
```

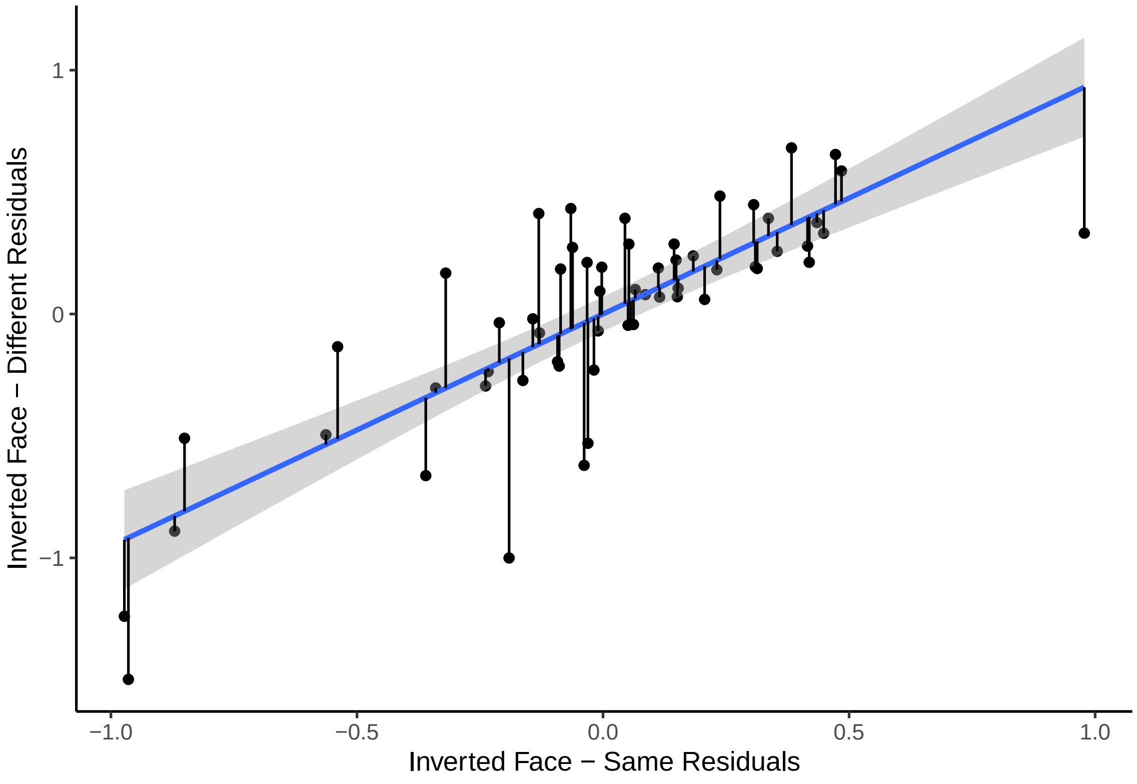

Contrast Detection - Different Residuals ~ Same Residuals.

```
##
## Call:
## lm(formula = lidresid ~ lisresid, data = b3)
##
## Coefficients:
## (Intercept)     lisresid
##   1.051e-17    1.092e-01
```

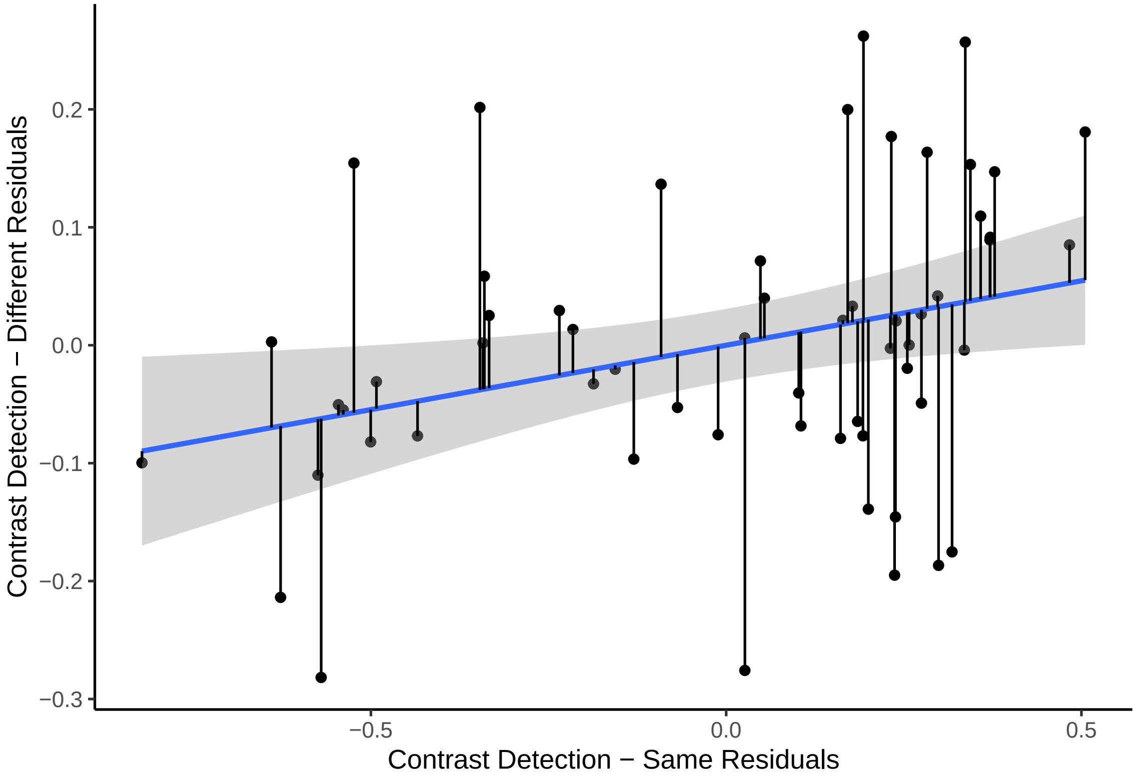

Supplement: S4 File — Models used for the regression-of-regressions method. (PDF) [file pone.0285255.s004.pdf]
