# Supplementary material for: Contributions of low- and high-level contextual mechanisms to human face perception
Source: PLoS One. 2023 May 2;18(5):e0285255. doi: 10.1371/journal.pone.0285255 (PMC10153715; doi:10.1371/journal.pone.0285255)
Supplement: S5 File — The details of the model selection procedure is explained in this file. (PDF) [file pone.0285255.s005.pdf]

SUPPLEMENTARY 5 - Contributions of low- and high-level contextual mechanisms to human face perception Model Comparisons

M. Umut Canoluk[1], Pieter Moors[2], & Valerie Goffaux[1,3,4]

[1] Research Institute for Psychological Science, Université Catholique de Louvain, Louvain-la-Neuve, Belgium
[2] Brain and Cognition, KU Leuven, Leuven, 3000, Belgium
[3] Department of Cognitive Neuroscience, Maastricht University, Maastricht, the Netherlands
[4] Institute of Neuroscience (IoNS), University of Louvain, Louvain-la-Neuve, Belgium

Contents

1 Few notes on BRMS formula syntax **2**

2 Models included in the comparison and their respective formulae **2**

2.1 Model 1 2

2.2 Model 2 2

2.3 Model 3 3

2.4 Model 4 3

2.5 Model 5 3

3 Model comparison using leave-one-out (LOO) cross-validation **4**

References **4**

1 Few notes on BRMS formula syntax

In BRMS formula syntax, the variable before the ~ sign relates to the dependent variable.

Anything on the right side of the ~ sign specifies independent variables. The plus (+) sign is used to separate different main effects from one another. The asterisk (*) defines interactions between independent variables.

Lastly, random effect structure can be defined using bars (||), where one or more variables are assumed to vary with the levels of the grouping factor defined after the bar. In our case, this factor is "subject", as we expect the thresholds to vary between subjects. More information about the brms syntax can be found in Bürkner (2018).

2 Models included in the comparison and their respective formulae

We tested five models that would provide thresholds for the purposes of our analyses before choosing the ideal one. Below we report the formula of each model. In the formula, "iv3" refers to the local input strength, which is our common independent variable for the both tasks. This represents contrast in the contrast detection task, and dissimilarity in the eye matching task. "cond" refers to context conditions (Same, Different, Isolated) and task defines which task the data belonged to.

2.1 Model 1

Model in which task (Upright Face, Inverted Face, Contrast Detection) does not interact with other variables.

```
## dv ~ 0.5 * guess + (1 - 0.5 * guess) * inv_logit(eta)
## eta ~ iv3 * cond + task + (iv3 * cond + task || subject)
```

2.2 Model 2

Model in which local input strength does not interact with other variables.

```
## dv ~ 0.5 * guess + (1 - 0.5 * guess) * inv_logit(eta)
## eta ~ iv3 + cond * task + (iv3 + cond * task || subject)
```

2.3 Model 3

Model in which no variable interacts with one another, and only main effects included.

```
## dv ~ 0.5 * guess + (1 - 0.5 * guess) * inv_logit(eta)
## eta ~ iv3 + cond + task + (iv3 + cond + task || subject)
```

2.4 Model 4

Model with complete interaction between all variables, without random effect structure.

```
## dv ~ 0.5 * guess + (1 - 0.5 * guess) * inv_logit(eta)
## eta ~ iv3 * task * cond
```

2.5 Model 5

Model with complete interaction between all variables, and with random effect structure.

```
## dv ~ 0.5 * guess + (1 - 0.5 * guess) * inv_logit(eta)
## eta ~ iv3 * task * cond + (iv3 * task * cond || subject)
```

3 Model comparison using leave-one-out (LOO) cross-validation

Following Vehtari, Gelman, and Gabry (2017), we compared these models using LOO comparison method. A model fit can be measured by calculating the Watanabe-Akaike Information Criterion (WAIC). A lower WAIC points to a better fitting model (Kurz, 2019; Vehtari et al., 2017). LOO cross-validation allows to compare these values and plot them. Model 5 had the lowest WAIC (75334 ± 331), therefore was the ideal one (see Figure S.4 for its ranking against other models).

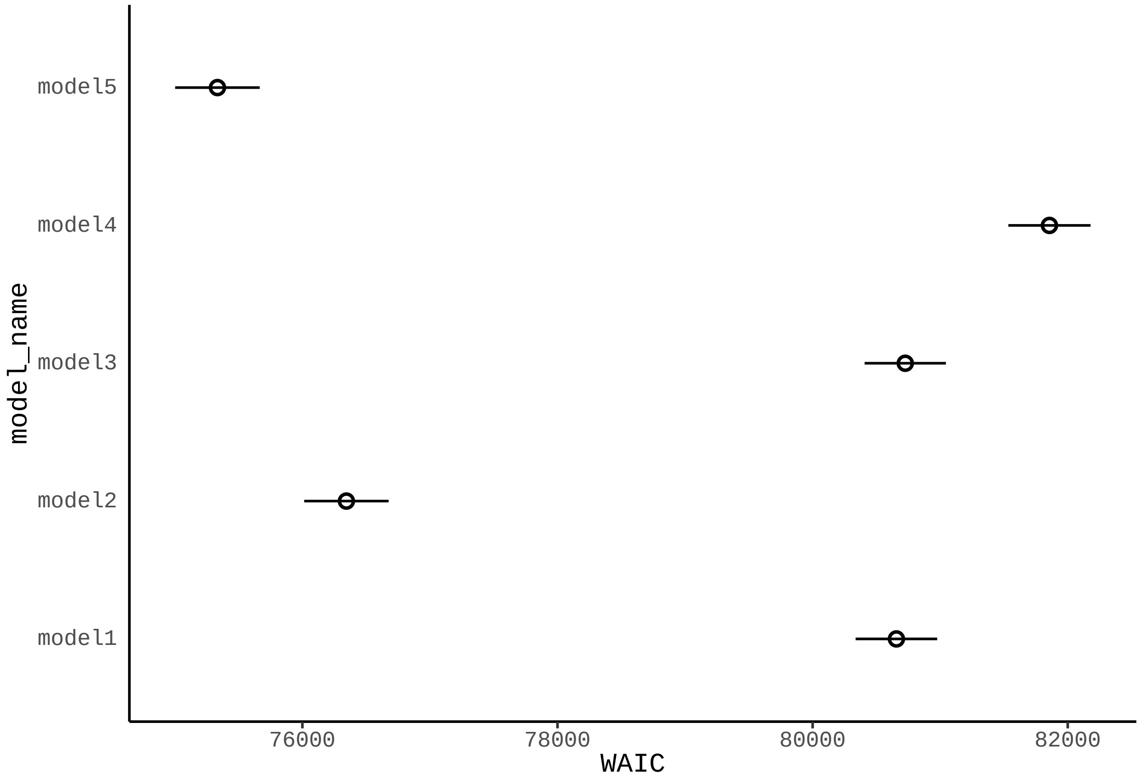

Figure S.4: As can be seen in the figure here, Model 5, (the complete interaction model with random effect structure) has the lowest WAIC, hence is the best fitting model for the data. Therefore, we decided to use this model to obtain thresholds for further analyses. Points represent WAIC values, error bars represent standard errors.

References

Bürkner, P.-C. (2018). Advanced Bayesian Multilevel Modeling with the R Package brms. *The R Journal*, *10*(1), 395. https://doi.org/10.32614/RJ-2018-017

Kurz, S. (2019). *Statistical Rethinking with brms, Ggplot2, and the tidyverse.* (Retrieved from osf.io/97t6w).

Vehtari, A., Gelman, A., & Gabry, J. (2017). Practical Bayesian model evaluation using leave-one-out cross-validation and WAIC. *Statistics and Computing*, *27*(5), 1413–1432. https://doi.org/10.1007/s11222-016-9696-4
